# Myeloma cell-derived CXCL7 facilitates proliferation of tumor cells and occurrence of osteolytic lesions through JAK/STAT3 pathway

**DOI:** 10.1038/s41419-025-07413-6

**Published:** 2025-02-06

**Authors:** Yue Wang, Tianwei Lan, Qiongyan Zhang, Chi Zhou, Peng Liu

**Affiliations:** 1 Department of Hematology, Zhongshan Hospital, Fudan University, Shanghai, China; 2 Department of Pathology, Zhongshan Hospital, Fudan University, Shanghai, China

**Keywords:** Myeloma, Oncogenes

## Abstract

Osteolytic lesions and pathological fractures are hallmark manifestations of multiple myeloma (MM), profoundly influencing the quality of life and self-care ability of MM patients. By analyzing transcriptome data and single-cell RNA-seq data from our center and the GEO database, a subset of MM cells with high expression levels of chemokine CXCL7 was identified. This subset of MM cells possesses a high capacity for proliferation and activation of osteoclast signaling pathway. CXCL7 might be a crucial regulator of osteolytic damage in MM. Subsequently, the association between the expression level of CXCL7 and pathological fractures in patients was investigated, and the impact of CXCL7 on MM proliferation was confirmed both in vivo and in vitro. A mouse xenograft tumor model was established through intravenous injection of myeloma cell lines based on the homing ability of plasma cells. Moreover, the mechanism by which CXCL7 promotes the activation of osteoclast signaling pathways in MM was explored. Our findings reveal that elevated CXCL7 levels significantly enhance MM cell proliferation, increasing the risk of pathological fractures in MM patients. Additionally, our mouse xenograft tumor model demonstrated that CXCL7 can induce femoral fractures and reduce bone mineral density. Concurrently, it was discovered that CXCL7 could activate the JAK/STAT3 pathway via CXCR2 and upregulate the expression levels of MMP13 and C-myc, facilitating MM cell proliferation and activation of the osteoclast signaling pathway. Our study offers novel insights into the pathogenic mechanism of osteolytic lesions and implies that targeting CXCL7 may present a new therapeutic avenue for MM.

## Introduction

Multiple myeloma (MM) is a difficult-to-cure malignant tumor with a relatively slow progression and high intra-tumor heterogeneity [1]. MM presents with diverse clinical manifestations, target organ damage, and prognosis among different patients [2]. The emergence of osteolytic lesions and the consequent occurrence of pathological fractures represent one of the crucial factors affecting the quality of life and prognosis of patients with myeloma. Pathological fractures also serve as a significant cause for patients with myeloma to lose their self-care capacity in the terminal stage [3, 4]. Osteolytic injury in myeloma is driven by two key factors. First, the proliferation of myeloma cells suppresses normal bone marrow function. Second, the interaction between myeloma cells and the bone marrow microenvironment activates osteoclast-related pathways, leading to osteolytic damage [5]. Patients with MM often experience relatively long overall survival. In fact, as opposed to the complete eradication of monoclonal plasma cells, modulating the biological behavior of myeloma cells to extend the survival period with tumor and concurrently control the degree of target organ damage could be a more efficacious therapeutic modality. Identifying key genes that influence myeloma cell behavior, particularly those involved in regulating osteoclast signaling, is therefore of significant clinical importance.

Through a comprehensive analysis of RNA-seq and single-cell sequencing data of myeloma, we identified that in myeloma, CXCL7 has the capacity to facilitate osteolytic destruction and the proliferation of myeloma. Chemokine CXCL7 pertains to a category of chemotactic cytokines and assumes a crucial role in regulating the migration and functionality of immune cells [6]. In tumors, chemokines not only direct the migration of immune cells to influence the anti-tumor immune response [7] but also regulate the proliferation, invasion, metastasis, and angiogenesis of tumor cells by activating diverse signaling pathways such as STAT3 and NF-κB [8, 9]. However, the exact mechanisms by which CXCLs influence tumors remain unclear. Plasma cells, derived from B cells, are influenced by chemokines that regulate their infiltration, proliferation, and interactions with stromal cells in tissues, affecting their overall biological functions. That is to say, chemokines might play a decisive role in immune cell tumors. Previous studies have demonstrated that CXCL7 exerts a role in promoting proliferation and invasion in malignant tumors such as breast cancer, lung cancer, and renal cancer, and is associated with an adverse prognosis [10–12]. However, the role of CXCL7 in MM remains indistinct. This study aims to explore the role and related mechanisms of CXCL7 in MM to furnish a theoretical basis for identifying novel therapeutic targets.

## Results

### CXCL7 is one of the key genes associated with pathological fractures in myeloma

**Fig. 1:**
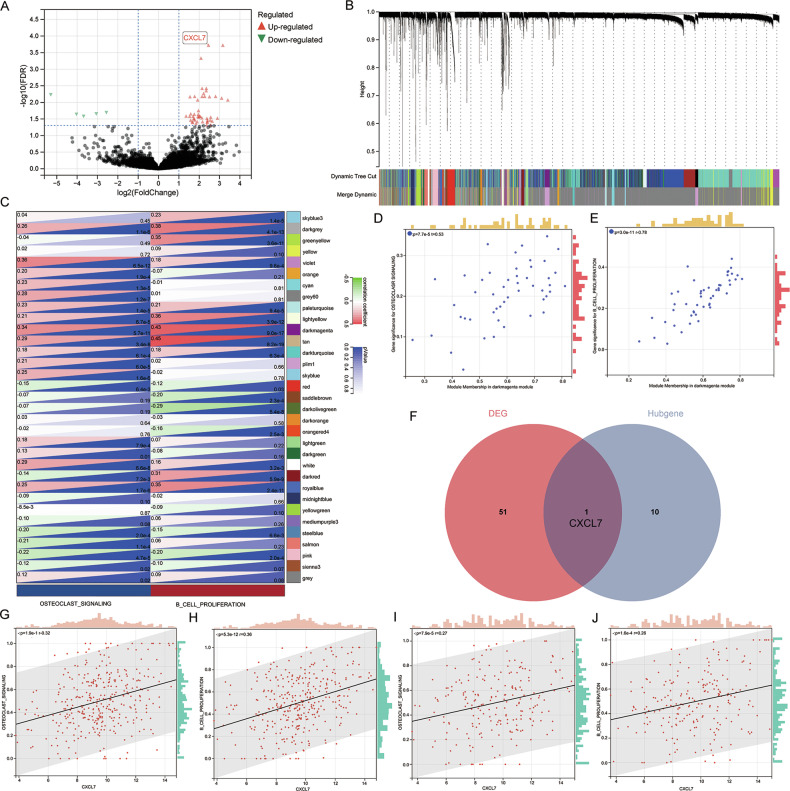
CXCL7 plays a crucial role in the proliferation of multiple myeloma and the activation of osteoclast signaling pathways. **A** The volcano plot of differentially expressed genes in the plasma cells from 15 MM patients with pathological fracture and 15 MM patients without pathological fracture (|logFC | > 1, FDR < 0.05). **B** Results of weighted gene co-expression network analysis of RNA-seq data in the TT2 set. **C** Correlation between different co-expression modules and “Osteolysis signaling“ and “B cell proliferation”. The relationship between genes in the dark magenta co-expression module and “Osteolysis signaling”(**D**)/“B cell proliferation”(**E**) pathway. **F** A total of 11 hub genes related to osteoclast signaling pathways and B cell proliferation were discovered in the TT2 cohort. A total of 52 differentially expressed genes in the plasma cells of 30 patients with/ without pathological fracture were determined. The intersection gene was CXCL7. The expression level of CXCL7 and the activation of “Osteolysis signaling”(**G**)/“B cell proliferation”(**H**) pathway in TT2 set. The expression level of CXCL7 and the activation of “Osteolysis signaling”(**I**)/“B cell proliferation”(**J**) pathway in TT3 set).

Our research aimed to identify key genes affecting MM cell proliferation and osteoclast signaling. We collected bone marrow specimens from 30 newly diagnosed MM (NDMM) patients: 15 with more than five osteolytic lesions and pathological fractures, and 15 without osteolytic damage. After CD138 magnetic bead sorting, we performed high-throughput transcriptome sequencing, identifying 52 differentially expressed genes (|logFC | > 1, FDR < 0.01) (via DESeq2) (Fig. 1A).

Next, we analyzed the GSE24080 dataset, which includes two cohorts of NDMM patients: the Total Therapy 2 [13] (TT2) cohort (340 cases) and the Total Therapy 3 [14] (TT3) cohort (214 cases). TT2 cohort was designated as a training set, while TT3 cohort was utilized as a validation set. WGCNA analysis categorized the 23,517 genes in the TT2 set into 33 modules (Fig. 1B). Subsequently, ssGSEA was adopted to quantify the activation of “B cell proliferation” and “osteoclast signaling pathway” for all patients. Correlations between the co-expressed gene modules from WGCNA and pathway activation were examined. The results demonstrated that the dark magenta module significantly facilitated the activation of the “B cell proliferation” (*P* = 5.6e−11) and “osteoclast signaling” (*P* = 9.0e−17) pathways (Fig. 1C). Then, the correlations of all genes within the dark magenta module with these two traits were computed (Fig. 1D–E). Based on the truncation criterion (|Module membership | > 0.70), 11 genes were identified as hub genes. By intersecting the differentially expressed genes and the hub genes, we found that CXCL7 was a potential key regulator (Fig. 1F).

Further analysis in the TT2 cohort confirmed that CXCL7 expression was positively correlated with both “B cell proliferation” (*P* < 0.0001) (Fig. 1G) and “osteoclast signaling” (*P* < 0.0001) (Fig. 1H). This correlation was also validated in the TT3 cohort (Fig. 1I–J).

### MM cells featuring a high expression level of CXCL7 possess enhanced osteoclast signaling pathway activity

**Fig. 2:**
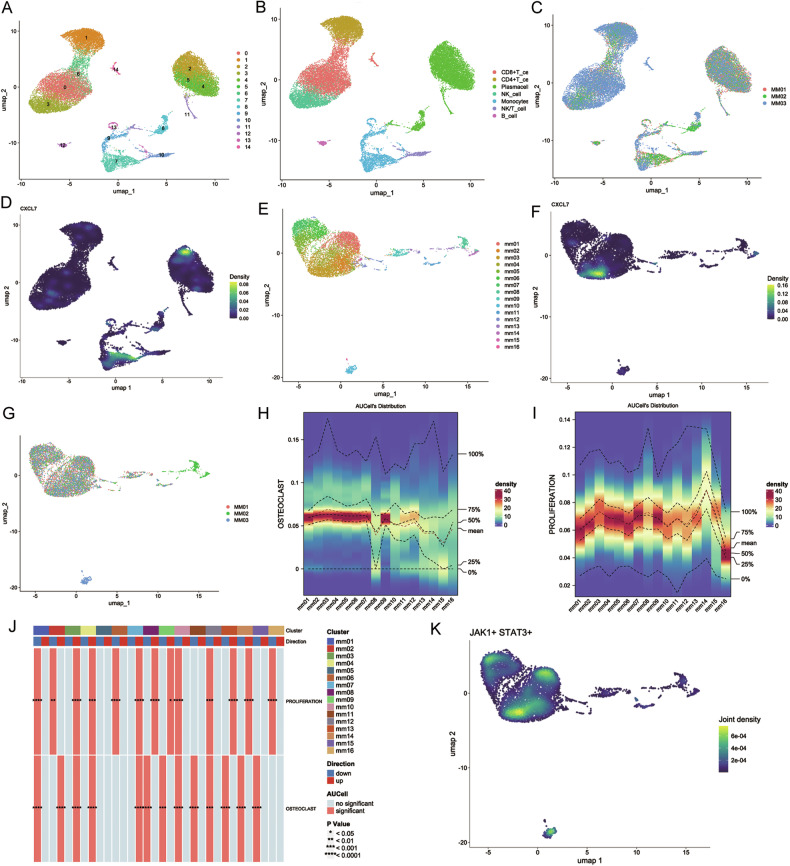
Single-cell RNA-seq findings confirmed that the activation intensities of the osteoclast signaling pathway and proliferation signaling pathway in the MM cell subpopulation with high expression of CXCL7 were more prominent. The samples were obtained from the bone marrow of three initially diagnosed multiple myeloma (MM) patients with pathological fractures. The three patients were respectively designated as MM01, MM02 and MM03. **A** Cell clustering results from single-cell RNA-seq in three newly diagnosed MM patients. **B** The cell subpopulation profiles of bone marrow samples from three patients with NDMM. **C** The distribution of bone marrow samples from three NDMM patients. **D** Expression of CXCL7 in all cells. **E**. Cell clustering results from single-cell RNA-seq of MM cells. **F** CXCL7 was mainly expressed in mm03 subgroup. **G** The distribution of MM cells among different patients. Activation level of the “Osteoclast signaling” (**H**) and “B-cell proliferation” (**I**) pathway in various MM subsets. **J** The mm03 subset exhibited a strong correlation with the “B cell proliferation” and “osteoclast signaling” pathways. **K** Activation status of the “JAK/STAT3” pathway in MM cells. In the mm03 subpopulation, the JAK/STAT3 pathway was strikingly activated. Additionally, a significant co-localization of the expression of CXCL7 and JAK/STAT3 was discerned).

Given the high intratumor heterogeneity of MM, we aim to further explore the characteristics of MM cell subsets with elevated CXCL7 expression. Bone marrow samples were collected from three NDMM patients with pathological fractures for single-cell RNAseq. The data from individual cells across all patients were integrated and analyzed using UMAP downscaling (Fig. 2A–C). In addition to high CXCL7 expression in monocytes, we observed a significant upregulation of CXCL7 in some plasma cells as well (Fig. 2D). Further analysis revealed 16 distinct clonal subpopulations of MM cells (Fig. 2E). CXCL7 expression was most prominent in the mm03 subgroup (Fig. 2F). Although CXCL7 is expressed only in a subset of MM cells, subgroup cells with an elevated CXCL7 expression can be identified in all three patients (Fig. 2G).

We selected “B cell proliferation” and “osteoclast signaling” as target pathways and conducted signaling pathway enrichment analysis. The results showed that the mm03 subgroup had the highest median activation of the “osteoclast signaling” pathway, with “B cell proliferation” ranking second (Fig. 2H–I). Moreover, the mm03 subgroup demonstrated the most significant correlation with both pathways (Fig. 2J).

### CXCL7 is associated with pathological fractures in patients

**Fig. 3:**
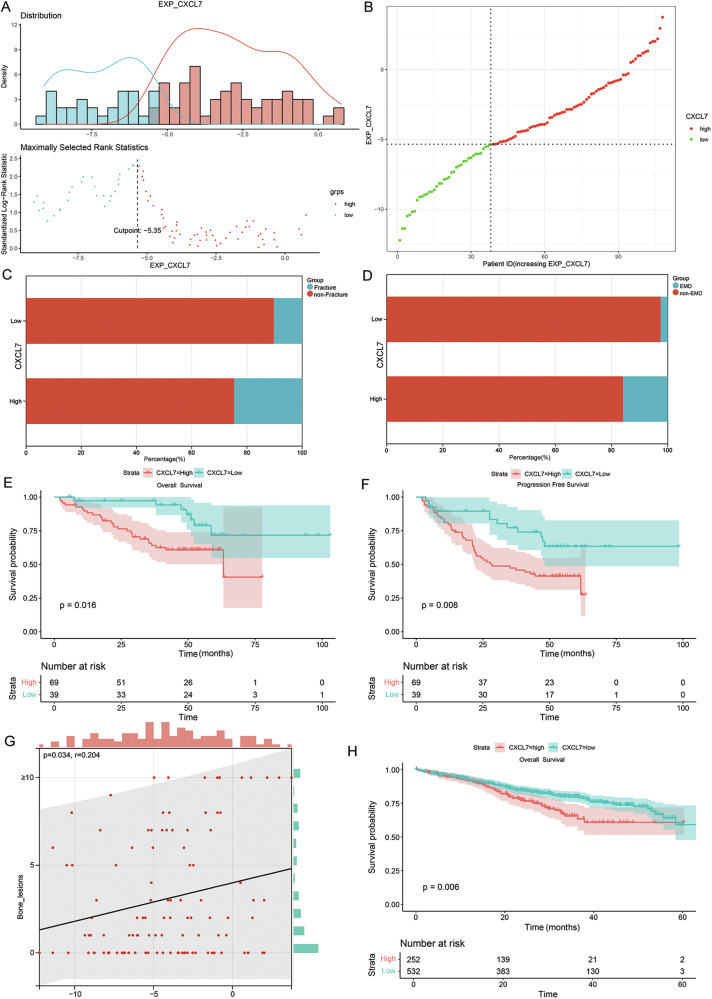
Prognostic implications and clinicopathological characteristics of patients stratified based on varying levels of CXCL7 expression. **A** The optimal cut-off value of the relative expression level of CXCL7. **B** The scatter plot illustrates the distribution of patient groups in relation to the gene expression levels of CXCL7. **C** Probability of pathological fracture in patients with different CXCL7 expression levels. **D** Probability of extramedullary invasion in patients with different CXCL7 expression levels. **E** Survival curves for overall survival time of patients in different CXCL7 groups. **F** Survival curves for progression-free-survival time of patients in different CXCL7 groups. **G** Correlation between the relative expression level of CXCL7 and the number of osteolytic bone lesions detected by PET-CT in 108 patients. **H** Survival curves for overall survival time of patients in different CXCL7 groups in the MMRF database).

We collected bone marrow samples from 108 NDMM patients from 2016 to 2020 and assessed CXCL7 expression using qPCR. All patients received an initial combination treatment regimen of proteasome inhibitors, immunomodulators, and dexamethasone. We determined the optimal cut-off value for CXCL7 and compared the prognosis and clinicopathological disparities among patients with different expression levels of CXCL7 (Fig. 3A–B). The findings revealed that patients in the high CXCL7 expression group had a higher likelihood of pathological fracture (P = 0.048) (Fig. 3C) and extramedullary invasion (*P* = 0.010) (Fig. 3D). Moreover, elevated levels of CXCL7 were associated with reduced overall survival (OS) (P = 0.016) and progression-free survival (PFS) (P = 0.008) (Fig. 3E–F). However, no significant correlation was identified between CXCL7 and ISS stage or high-risk cytogenetics. The clinical baseline characteristics of the patients are presented in Table 1. We then investigated the association between CXCL7 expression levels and the number of osteolytic lesions in patients. Osteolytic lesions were evaluated via PET-CT. Spearman correlation analysis revealed a significant positive correlation between CXCL7 expression and the number of osteolytic lesions (*P* = 0.034) (Fig. 3G).

**Table 1:** Baseline characteristics of 108 patients with multiple myeloma.

Variable	Total	CXCL7_High	CXCL7_Low
Patinets (No)	108	69	39
Gender (No,%)			
Male	74 (68.5)	46 (66.7)	28 (71.8)
Female	34 (31.5)	23 (33.3)	11 (28.2)
Age (year, (No, %))			
<60	30 (27.8)	15 (21.7)	15 (38.5)
≥60	78 (72.2)	54 (78.3)	24 (61.5)
ISS stage (No,%)			
I	43 (39.8)	29 (42.0)	14 (35.9)
II	28 (25.9)	16 (23.2)	12 (30.8)
III	37 (34.3)	24 (34.8)	13 (33.3)
FISH high-risk (No,%)			
Detected	34 (31.5)	23 (33.3)	11 (28.2)
Not detected	74 (68.5)	46 (66.6)	28 (71.8)
Follow-up time (month, (median, range))	48.7 (2.1–103.0)	46.3 (2.1–77.6)	52.2 (5.4–103.0)
Progress (No,%)	53 (49.1)	41 (68.1)	12 (30,8)
Dead (No,%)	34 (31.5)	27 (39.1)	7 (17.9)

To further validate the clinical relevance of CXCL7, we assessed its prognostic value in the Multiple Myeloma Research Foundation (MMRF) database, the largest publicly available dataset of MM patients. A total of 784 NDMM patients with complete prognostic data and RNA-seq data were included in the study. Survival analysis, based on the optimal cutoff value, revealed that higher CXCL7 expression was significantly associated with shorter OS time (P = 0.006) (Fig. 3H).

### CXCL7 promotes proliferation of MM cells

**Fig. 4:**
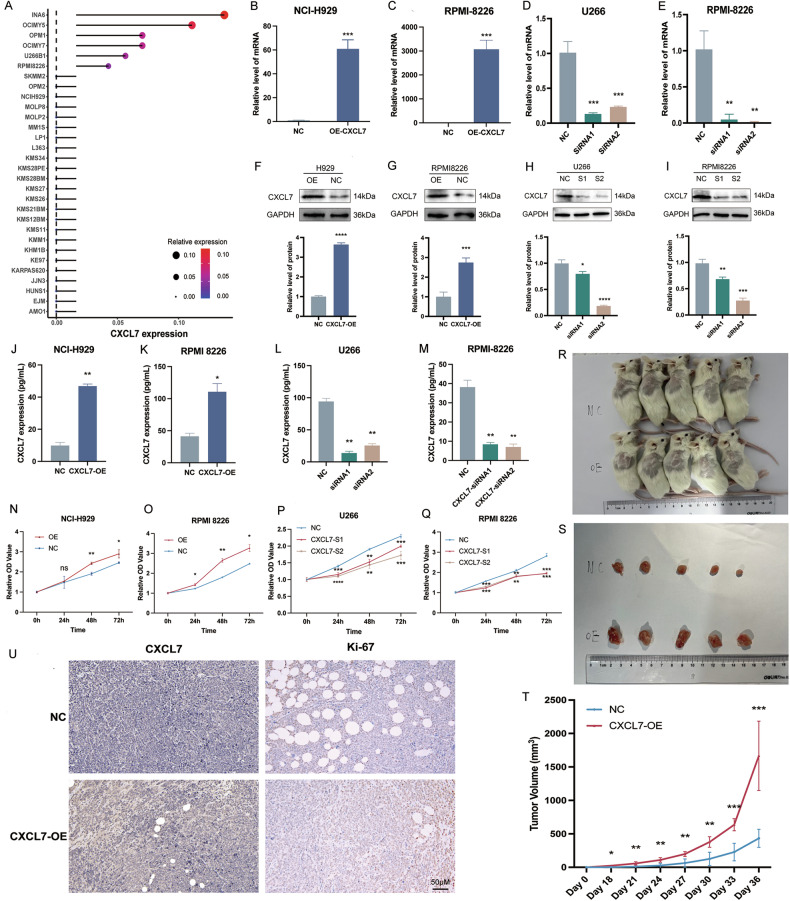
CXCL7 facilitates the proliferation, migration and invasion of MM cells. **A**. The expression level of CXCL7 in MM cell lines within the CCLE database. The expression level of CXCL7 in the CXCL7-OE and NC H929 (**B**)/RPMI 8226 (**C**) cell lines was evaluated via qPCR. The expression level of CXCL7 in the CXCL7-KD and NC U266(**D**)/RPMI 8226 (**E**) cell lines was evaluated via qPCR. Western blot analysis of the expression level of CXCL7 protein in the CXCL7-OE and NC H929(**F**)/RPMI8226 (**G**) cell lines. Western blot analysis of the expression level of CXCL7 protein in the CXCL7-KD and NC U266 (**H**)/RPMI8226 (**I**) cell lines. ELISA was utilized to assess the levels of CXCL7 extracellular secreted protein in the CXCCL7-OE and NC H929 (**J**)/RPMI8226 (**K**) cell lines. ELISA was utilized to assess the levels of CXCL7 extracellular secreted protein in the CXCCL7-KD and NC U266 (**L**)/RPMI8226 (**M**) cell lines. **N**–**Q** The CCK8 assay was employed to assess the differences in cell proliferation rates among CXCL7-OE, CXCL7-KD, and NC group. **R**, **S** Subcutaneous xenograft tumors of mice in CXCL7-OE group and NC group. **T** The mean growth rate of subcutaneous xenograft tumors in CXCL7-OE and NC mice. **U** The expression levels of CXCL7 and Ki-67 in the subcutaneous xenograft tumors of the CXCL7-OE group and NC group (via immunohistochemical).

In CCLE database, CXCL7 expression was found to be low in the NCI-H929 cell line, moderate in the RPMI 8226 cell line, and high in the U266 cell line (Fig. 4A). Lentiviruses carrying CXCL7-overexpression (CXCL7-OE) and Normal-Control (NC) were used to infect NCI-H929 and RPMI 8226 cell lines. Meanwhile, siRNA was employed for CXCL7 knockdown (CXCL7-KD) in U266 and RPMI 8226 cell lines. CXCL7 RNA levels were measured by qPCR (Fig. 4B–E). Protein expression, both intracellular and secreted, was assessed using Western blotting (Fig. 4F–I) and ELISA (Fig. 4J–M).

The CCK-8 assay was used to evaluate cell proliferation. The results showed a significantly higher proliferation rate in the CXCL7-OE group for both H929 and RPMI 8226 cell lines (Fig. 4N–O). In contrast, the CXCL7-KD group exhibited slower proliferation in U266 and RPMI 8226 cells (Fig. 4P–Q). Subcutaneous xenograft tumor models were established in B-NGD mice with CXCL7-OE and NC RPMI 8226 cell lines. Analysis revealed accelerated tumor growth in the CXCL7-OE group (Fig. 4R–T). Immunohistochemistry performed on excised tumors demonstrated elevated levels of Ki-67 proliferation index in the CXCL7-OE group (Fig. 4U).

### CXCL7 facilitate incidence of pathological fractures in mouse

**Fig. 5:**
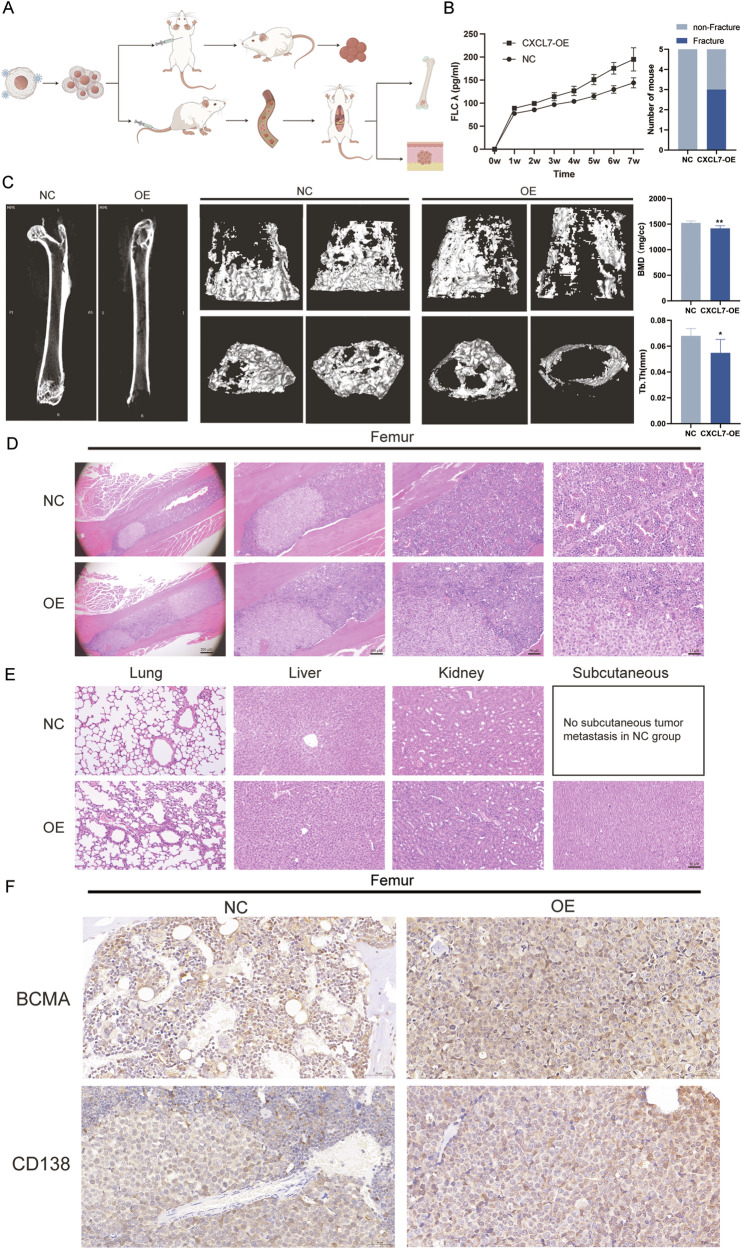
Impact of CXCL7 on the metastatic potential of MM cells in vivo. **A** Schematic diagram of subcutaneous xenograft model and tail vein injection xenograft model in mice (the image materials originated from Figdraw). **B** Quantitative levels of free λ light chain in the veins of CXCL7-OE and NC group; as well as the ratios of pathological fractures in mice of different groups. **C** The micro-CT scanning profiles of the leg bones in distinct groups of mice were presented. We exhibited the CT scan images of the femurs, along with the images of bone mineral density and trabecular bone of representative mice in the NC group and the OE group. The outcomes revealed that the bone mineral density (BMD) of the femurs in the CXCL7-OE group of mice was significantly lowered (*P* = 0.007), and the average trabecular thickness (Tb, Th) was significantly diminished (*P* = 0.037). **D** The presence of tumor colonization in the femur was confirmed through HE staining in both the CXCL7-OE group and the NC group. The tumor burden within the femur of mice in the NC group was more considerable. **E** HE staining was employed to identify the presence of lung, liver, kidney, and subcutaneous tumor colonization in mice from the CXCL7-OE group and NC group. There were no significant disparities in the lung, liver, and kidney lesions between the mice in the OE group and those in the NC group. However, subcutaneous soft tissue metastatic foci were identified in the mice of the OE group, but not in those of the NC group. **F** BCMA and CD138 were marked by immunohistochemistry for the evaluation of the occurrence of monoclonal plasma cells in the femoral bones of mice within the OE and NC cohorts. Evident plasma cell infiltrations were exhibited in the bone marrow of both cohorts).

Subsequently, a murine xenograft model was established via intravenous infusion of MM cells to scrutinize the colonization of CXCL7-OE MM cells in the bone marrow by virtue of the homing effect and its ramification on the occurrence of osteolytic lesions (Fig. 5A). This model mimics the in vivo behavior of plasma cells, allowing us to investigate the influence of CXCL7 on myeloma cell activity in a more physiologically relevant context. At 7 weeks, three out of five mice in the CXCL7-OE group exhibited symptoms of long bone fractures. We performed regular venous blood collection to measure serum free light chain levels and assess tumor burden, which showed a significantly higher tumor burden in the CXCL7-OE group (Fig. 5B).

Micro-CT imaging of femoral bones revealed more pronounced bone deterioration in the CXCL7-OE group (Fig. 5C). Specifically, bone mineral density (BMD) was significantly reduced in the CXCL7-OE group (*P* = 0.007), with a mean decrease of 105 mg/cc compared to the NC group. Additionally, the average trabecular thickness (Tb, Th) was significantly decreased in the CXCL7-OE group (P = 0.037), indicating that CXCL7 overexpression accelerates bone loss and disrupts trabecular structure.

Then, tissue samples from the femur, subcutaneous metastases, livers, lungs, and kidneys were collected for histopathological examination via hematoxylin and eosin (HE) staining (Fig. 5D–E). Subsequently, we performed immunohistochemical analysis to detect BCMA and CD138, which allowed us to assess the presence of monoclonal plasma cells in the bone marrow of mice. Distinct BCMA (+) and CD138 (+) plasma cell infiltrates were observed in the femurs of both the OE and NC groups (Fig. 5F). The femur sections revealed that myeloma cells in the CXCL7-OE group showed enhanced colonization and homing abilities within the bone marrow.

### CXCL7 activates the JAK/STAT3 signaling pathway

**Fig. 6:**
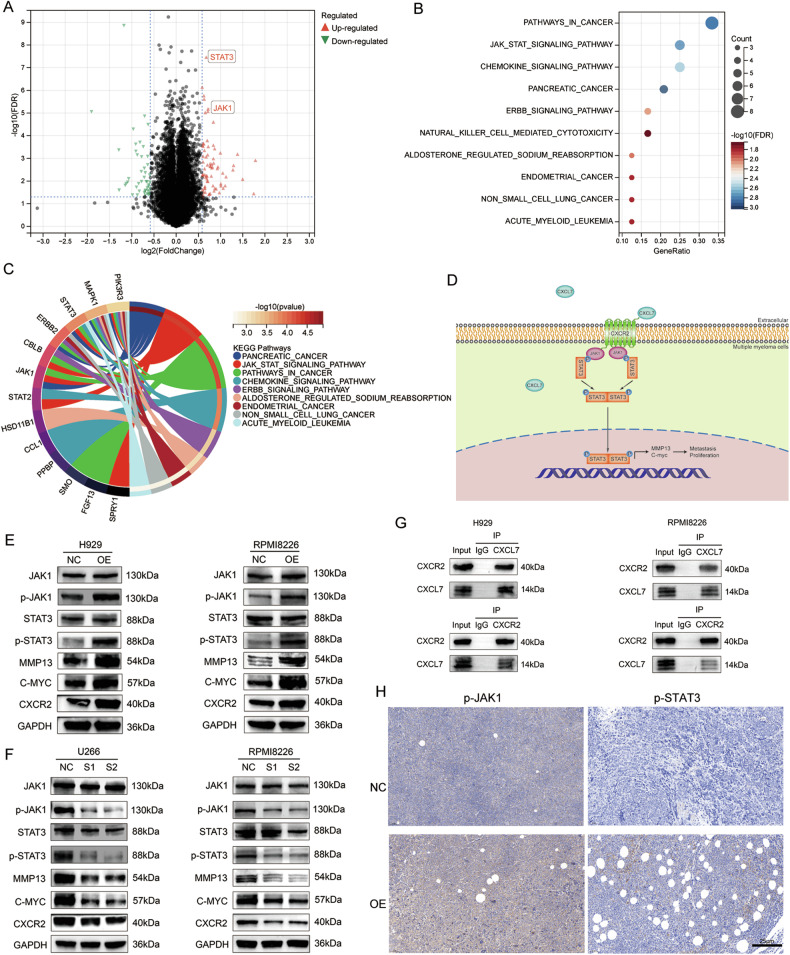
CXCL7 binds to CXCR2 through autocrine of MM cells and activates the intracellular JAK/STAT3 signaling pathway. **A** The volcano plot of the differentially expressed genes revealed by RNA-seq profiling of the CXCL7-OE and NC RPMI8226 cell lines (|logFC | > 1, *p* < 0.05). **B**, **C** Enrichment analysis of KEGG pathways for differentially expressed genes in the CXCL7-OE and NC RPMI8226 cell lines. **D** The hypothesized mechanism schema of how CXCL7 affects intracellular signaling pathways in MM cells: CXCL7, autocrined by MM cells, binds to CXCR2, thereby activating the JAK/STAT3 pathway and further up-regulating MMP13 and C-myc. Western blot analysis was conducted to evaluate the protein expression levels of JAK1, p-JAK1, STAT3, p-STAT3, MMP13, C-myc and CXCR2 in H929, U266, and RPMI 8226 cells within the CXCL7-OE group, CXCL7-KD group, and NC group. In the CXCL7-OE group, a significant upregulation of the JAK/STAT3 pathway, MMP13, C-myc and CXCR2 was observed (**E**); while in the CXCL7-KD group, a notable downregulation was witnessed (**F**). **G** Co-immunoprecipitation (Co-IP) assay was performed to confirm the combination between CXCL7 and CXCR2 in H929 and RPMI8226 cells. **H** In the mouse xenograft model, immunohistochemistry results revealed a significant upregulation of JAK/STAT3 in the tumors of the CXCL7-OE group).

We conducted RNA-seq on CXCL7-OE and NC RPMI 8226 cell lines and identified differentially expressed genes (Fig. 6A). KEGG pathway analysis revealed significant enrichment of the JAK/STAT signaling pathway in the CXCL7-OE cells (Fig. 6B–C). Among the differentially expressed genes, JAK1 and STAT3 were conspicuously upregulated. Prior studies have validated that CXCR2 acts as the primary receptor of CXCL7, and the JAK/STAT3 is one of the pathways activated following the activation of CXCR2 [15, 16]. We postulate that CXCL7 activates the JAK/STAT3 pathway via CXCR2, thereby influencing the proliferation of MM cells and further triggering the expression of downstream activating proteins associated with the osteoclast signaling pathway (Fig. 6D).

To explore the JAK/STAT3 signaling pathway, we carried out WB to evaluate the expression of JAK1, phosphorylated JAK1 protein (p-JAK1), STAT3, phosphorylated STAT3 (p-STAT3) and CXCR2 in different MM cell lines. In both H929 and RPMI 8226 cell lines, the levels of p-JAK1 and p-STAT3 were significantly increased in CXCL7-OE group (Fig. 6E). Conversely, in U266 and RPMI 8226 cell lines, these levels were reduced in CXCL7-KD group (Fig. 6F). Additionally, the expression level of CXCR2 was significantly upregulated in CXCL7-OE H929 and RPMI8226 cells (Fig. 6E) and significantly downregulated in CXCL7-KD U266 and RPMI8226 cells (Fig. 6F). Co-immunoprecipitation (Co-IP) confirmed that CXCL7 interacts with CXCR2 in both H929 and RPMI8226 cells (Fig. 6G). These results suggest that increased CXCL7 expression enhances its binding to CXCR2 and activates the JAK/STAT3 pathway.

Immunohistochemical analysis of the mouse xenograft tumor model further confirmed significant JAK/STAT3 pathway activation in CXCL7-OE group (Fig. 6H). Additionally, single-cell RNA sequencing of MM patient samples revealed a strong co-expression of CXCL7 and JAK/STAT3 in the mm03 subgroup (Fig. 2K). JAK/STAT3 forms a crucial oncogenic pathway in MM, traditionally believed to be activated by IL-6. However, in the absence of IL6, most MM cell lines still have a highly active JAK/STAT3 pathway. Our research found that a subset of MM cells secreting CXCL7 can activate the JAK/STAT3 pathway through autocrine effects. Also, we observed enhanced activation of JAK/STAT3 in other cell subgroups (Fig. 2K), providing evidence for its potential activation via multiple mechanisms.

### CXCL7 upregulates the expression levels of MMP13 and C-myc via JAK/STAT3 pathway

We next investigated the downstream genes activated by the JAK/STAT3 pathway. Transcriptome sequencing of CXCL7-OE cells revealed significant upregulation of matrix metalloproteinase 13 (MMP13) and C-myc. Both MMP13 and C-myc are crucial effector proteins in response to JAK/STAT3 activation and are closely associated with osteoclast signaling and MM cell proliferation. Consequently, we proceeded with the validation of MMP13 and C-myc expression levels. The results showed a significant increase in both MMP13 and C-myc in CXCL7-OE cells (Fig. 6E), whereas their expression was significantly reduced in CXCL7-KD cells (Fig. 6F).

**Fig. 7:**
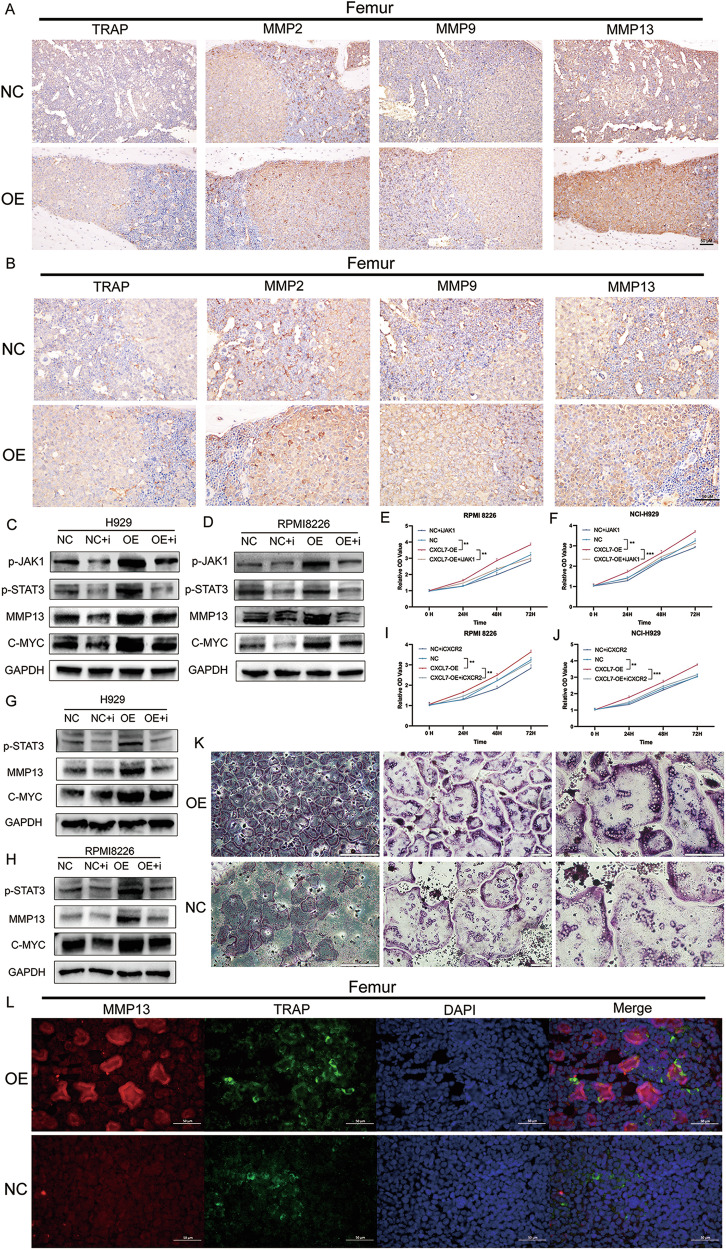
CXCL7 activates the JAK/STAT3 pathway, resulting in the upregulation of MMP13 and C-myc, further promoting osteoclast signaling pathway activation and tumor proliferation. **A**, **B** Immunohistochemical analyses were performed to assess the expression levels of TRAP, MMP2, MMP9, and MMP13 in the femurs of mice within the OE group and the NC group. TRAP was employed for labeling osteoclasts, and the results indicated a substantial increase in the quantity of osteoclasts in the OE group. MMP2, MMP9, and MMP13 were used to mark the relevant proteins related to the activation of osteoclast signaling pathways. The observations also revealed that the expression levels of MMP2, MMP9, and MMP13 in the bone tissue of mice in the OE group were significantly elevated. In the CXCL7-OE RPMI8226 (**C**) and H929 (**D**) cell lines, the incorporation of CXCR2 inhibitors incurs a significant downregulation of the JAK/STAT3 pathway, concurrently accompanied by a significant reduction in the expression magnitudes of MMP13 and C-myc. In the CXCL7-OE RPMI8226 (**E**) and H929 (**F**) cell lines, the administration of CXCR2 inhibitors elicited a significant attenuation of the cell proliferative capacity. In the CXCL7-OE RPMI8226 (**G**) and H929 (**H**) cell lines, the incorporation of JAK1 inhibitors gave rise to a significant reduction in the expression levels of p-STAT3, MMP13, and C-myc. In the CXCL7-OE RPMI8226 (**I**) and H929 (**J**) cell lines, the administration of JAK1 inhibitors elicited a significant attenuation of the cell proliferative capacity. **K** TRAP staining of CXCL7-OE/NC RPMI8226 cell lines co-cultured with primary mouse BMDMs. After 6 days, the CXCL7-OE group showed a higher number of multinucleated osteoclasts, indicating that CXCL7 promotes osteoclast differentiation. **L** Immunofluorescence was implemented for the precise detection of TRAP and MMP13, where TRAP acted as a definitive marker of osteoclasts. The obtained results manifested that in the CXCL7-OE group, TRAP and MMP13 presented conspicuous co-localization, inferring that MMP13 assumes a crucial role in the activation of osteoclasts.

Meanwhile, we utilized TRAP as a marker for osteoclasts and observed a significant increase in osteoclast numbers in the bone tissue of CXCL7-OE mice. Furthermore, we assessed the expression levels of MMP2, MMP9, and MMP13, which serve as markers for proteins associated with the activation of the osteoclast signaling pathway. Our findings revealed a significant elevation in the expression of all three MMPs in the bone tissue of the CXCL7-OE mice, with MMP13 showing the highest expression (Fig. 7A–B).

To further substantiate that the upregulation of JAK/STAT3 is attributable to CXCR2, we treated MM cells with SB225002, a CXCR2 inhibitor. This treatment led to a significant reduction in JAK/STAT3 activation (Fig. 7C–D) and cell proliferation (Fig. 7E–F). We further treated CXCL7-OE cells with Ruxolitinib (JAK inhibitor) (Fig. 7G–H). The results demonstrated that both p-STAT3 expression and cell proliferative capacity were significantly suppressed (Fig. 7G–J). Furthermore, treatment of MM cells with CXCR2 inhibitors and JAK inhibitors resulted in downregulation of MMP13 and C-myc expression (Fig. 7C–D & G–H).

We further explored the relationship between CXCL7 and the expression of CXCR2 and MMP13 using data from the MMRF database. Our analysis revealed a significant positive correlation between CXCL7 and both CXCR2 (P < 0.001) and MMP13 (P < 0.001) (Fig. S1).

### Elevated expression of CXCL7 in MM cells significantly facilitates the generation of osteoclasts

To further validate that high CXCL7 expression in MM cells promotes osteoclastogenesis, we co-cultured RPMI8226 CXCL7-OE/NC cell lines with bone marrow-derived macrophages (BMDMs) isolated from C57BL/6 mice. M-CSF and RANKL were added to the BMDMs to induce osteoclast differentiation. After 6 days of co-culture, TRAP staining was performed to assess osteoclast formation. The results showed that BMDMs co-cultured with CXCL7-OE cells generated a significantly higher number of multinucleated osteoclasts compared to the NC group, indicating that elevated CXCL7 expression enhances osteoclastogenesis (Fig. 7K).

Subsequently, immunofluorescence was employed to examine both MMP13 and TRAP (a marker for osteoclasts) within femoral bone marrow from mouse xenograft models (Fig. 7L). The results revealed a marked co-localization of MMP13 around TRAP-positive multinucleated osteoclasts, suggesting a strong interaction between MMP13 and osteoclasts. In contrast, no such co-expression was observed in the NC group, further supporting the role of MMP13 secreted by myeloma cells in facilitating osteoclast activity.

## Discussion

MM is a heterogeneous tumor characterized by diverse subpopulations of myeloma cells with distinct biological behaviors [5, 17, 18]. Osteolytic damage and pathological fractures are hallmark clinical symptoms of myeloma, leading to restricted mobility and loss of self-care ability in MM patients [4]. Our research identifies CXCL7 as a key gene contributing to pathological fractures in MM. Subpopulations of MM cells with elevated CXCL7 expression show enhanced activation of the osteoclast signaling pathway. Notably, CXCL7 is a member of the chemokine family, which plays a critical role in regulating immune cell function [19, 20]. Given that MM originates from lymphocytes, chemokines like CXCL7 are likely key regulators of myeloma cell behavior.

Current research on the role of CXCL7 in tumors is limited. Previous studies have shown that in glioma, tumor-induced macrophages secrete CXCL7 to enhance glioma stem cell functionality [21]. In breast cancer, targeting CXCL7 inhibits tumor growth and metastasis [10], while in cholangiocarcinoma, CXCL7 promotes tumor proliferation and invasion via the AKT signaling pathway [22]. In MM, CXCL7 has been identified as the predominant activator of MMP13. CXCL7 can be secreted by MSCs, thereby stimulating MM cells to produce a significant amount of MMP13, which constitutes a crucial means for activating the osteoclast signaling pathway [23, 24]. Our research has disclosed that in MM, not only stromal cells but also a subset of myeloma cells secrete CXCL7 in an autocrine manner. CXCL7 activates the JAK/STAT3 pathway within MM cells via the CXCR2 receptor and upregulates the expression of MMP13.

Through multiple analyses, we confirmed that MM cells with elevated CXCL7 expression secrete high levels of MMP13. A strong correlation was observed between the gene expression of CXCL7, CXCR2, and MMP13 in the MMRF database. However, no significant correlation was found between CXCL7 and MYC gene expression. We hypothesize that this lack of correlation arises because MMP13 in myeloma cells is primarily induced by CXCL7, whereas MYC, involved in various cancer pathways, is regulated by multiple mechanisms. As only a subset of myeloma cells secretes CXCL7, the potential association between CXCL7 and MYC expression may be diluted in the broader plasma cell RNA-seq data.

The JAK/STAT3 pathway is widely recognized as a crucial oncogenic pathway in MM. It was formerly hypothesized that it was primarily activated via IL6 [25]. Nevertheless, the activation of the JAK/STAT3 pathway is ubiquitous in MM [26]; numerous MM cells still exhibit a significantly activated JAK/STAT3 pathway in the absence of IL6, suggesting the involvement of alternative activation mechanisms. Our Single-cell RNA sequencing analysis revealed widespread activation of the JAK/STAT3 pathway across various MM subgroups, with a notable co-localization of JAK/STAT3 and CXCL7 in one subgroup. This concurrently implies that there are additional pathways for the activation of STAT3.

STAT3 is a key transcription factor involved in the initiation and progression of MM [27]. In our study, we observed that the upregulation of STAT3 significantly increased the protein levels of MMP13 and C-myc. MMPs play a critical role in osteolytic damage in MM and promote tumor growth by enhancing angiogenesis and facilitating the migration and invasion of MM cells [28–30]. Interestingly, previous studies have shown that IL6 can lead to the upregulation of MMP13, but CXCL7 is the most significant factor in upregulating MMP13 in MM [23, 24]. Our study explains this phenomenon, that is, CXCL7 leads to the upregulation of MMP13 through the activation of the JAK/STAT3 pathway.

Our research examined the role of CXCL7 in promoting osteolytic lesions in myeloma using various methods. In animal studies, we measured femur BMD using Micro-CT. BMD was calculated as Bone Mineral Content (BMC) divided by Bone Volume (BV). As BV was computed using a computerized method, fractured portions of the femur, which were disconnected from the rest of the bone, were excluded from the analysis. Given that the CXCL7-OE group exhibited more pathological fractures, this exclusion likely minimized differences in BDM between OE and NC groups. In the NC group, implantation of myeloma cells into the femurs also resulted in a decrease in BMD compared to normal mice. However, the OE group displayed a significantly greater reduction in BMD, with a decrease of over 100 mg/cc compared to the NC group, reflecting substantial bone loss.

Our findings highlight that a subset of myeloma cells secretes CXCL7 via an autocrine mechanism, playing a pivotal role in tumor progression and the development of osteolytic lesions. Targeting CXCL7 emerges as a promising therapeutic strategy, as inhibiting this autocrine loop could help prevent bone deterioration. Despite CXCL7 being expressed in only a fraction of myeloma cells, the high intra-tumor heterogeneity of myeloma suggests that targeting these particularly aggressive subpopulations could provide substantial clinical benefit. Blocking CXCL7 may reduce MMP13 production, decrease osteoclast activation, and mitigate bone resorption, ultimately lowering fracture risk and improving patient outcomes.

While research on CXCL7 inhibitors for MM is still in its early stages and no targeted drugs have been developed, the potential to treat patients with severe bone lesions is considerable. The development of CXCL7-targeted therapies could open new avenues for managing myeloma and its complications. A deeper understanding of CXCL7’s mechanisms, coupled with the development of effective inhibitors, will be essential for advancing therapeutic options and improving patient care.

## Methods and Materials

### Weighted correlation network analysis (WGCNA)

First, we utilized the gene expression profiles of patients in the GEO database. We chose the GSE24080 cohort and designated the TT2 patient group as the training set, while assigning the TT3 patient group as the validation set. In the TT2 training set, we separately calculated the Median Absolute Deviation (MAD) for each gene. To exclude the genes with the smallest MAD, we excluded the top 50% of genes. The R package “goodSamplesGenes” was utilized for the removal of outlier genes and samples, and WGCNA was further used to construct a scale-free co-expression network. Initially, we performed both Pearson’s correlation matrices and the average linkage method for all pairwise genes. After selecting the power of 5, the adjacency was transformed into a topological overlap matrix (TOM) to measure the network connectivity of a gene by defining it as the sum of its adjacency with all other genes for network gene ration, and the corresponding dissimilarity (1-TOM) was calculated. To classify genes with similar expression profiles into gene modules, we conducted average linkage hierarchical clustering according to the TOM-based dissimilarity measure with a minimum gene group size of 30 for the genes dendrogram. Additionally, we merged modules with a distance of less than 0.25.

### Single sample Gene Set Enrichment Analysis (GSEA)

Single sample GSEA (ssGSEA) analysis was performed to determine the degree of activation for each pathway, which was recorded as the Enrichment Score (ES). The score ranged from 0 to 1, indicating minimal to maximal activation. The activation scores for the “B cell tumor proliferation” pathway and the “osteoclast signaling activation” pathway were calculated using ssGSEA. The gene sets for these pathways were obtained from the MsiGDB database.

### The expression level of CXCL7 and clinicopathological features of samples

A total of 108 clinical samples of newly diagnosed MM patients were included in this study, which was collected from patients who visited Zhongshan Hospital of Fudan University from 2016 to 2020. In order to further observe the relationship between CXCL7 and the prognosis and drug treatment response of patients, only patients who had not undergone autologous hematopoietic stem cell transplantation were included. All patients received an initial treatment of a three-drug combination of protease inhibitor, lenalidomide, and dexamethasone. The bone marrow samples of patients were collected at the time of diagnosis. All bone marrow fluid samples were enriched with nucleated cells and sorted with CD138 magnetic beads. The expression level of CXCL7 is detected by the qPCR.

### Single cell mRNA sequencing

A diverse array of bone marrow samples was collected from Zhongshan Hospital affiliated with Fudan University, following approval from the Institutional Review Board of Zhongshan Hospital. Bone marrow mononuclear cells (BMMCs) were isolated and employed for single-cell RNA sequencing (scRNAseq). Single-cell libraries were generated from unsorted BMMCs using a 10× Genomics Chromium Single Cell 5′ Library Kit and Chromium instrument, followed by sequencing on an Illumina sequencer. The obtained FASTQ data was aligned to the reference genome (GRCh38) using Cell Ranger (v5.0.1), subsequently undergoing read filtering, barcode counting, and unique molecular identifier (UMI) counting. Seurat v4.0.1 software was utilized for cell clustering and dimension reduction. Single-cell data from different patients were integrated and analyzed via the UMAP downscaling approach. For pathway activation analysis in different cell subsets, we employed the irGSEA package along with AUCell and UCell methods to score pathway activation levels based on gene sets obtained from the MSigDB database.

### Cell culture

U266 and NCI-H929 cell lines are purchased from the Institute of Basic Medical Sciences, Chinese Academy of Medical Sciences, and RPMI-8226 cell line is obtained from the cell bank of the Chinese Academy of Sciences for use in this investigation. Cells are grown in RPMI 1640 media (Gibco, USA) with 10% fetal bovine serum (FBS) (Gibco, USA) under 37 °C, saturated humidity, and 5% CO2 conditions.

### Establishment of lentiviral stabilized cell lines

The CXCL7 overexpression and control lentiviruses were obtained from Genechem Biotechnology Co., Ltd. (Shanghai, China). The vector components are arranged in the following sequence: Ubi-MCS-3FLAG-CBh-gcGFP-IRES-puromycin. H929 and RPMI8226 cells were subjected to infection at a multiplicity of infection (MOI) of 100 for 12 h. Seventy-two hours post-transduction, the culture medium was amended with 2 μg/ml puromycin dihydrochloride (GeneChem, China) to facilitate the selection of stably transfected cells. The successful overexpression of CXCL7 was validated using Western blot analysis and quantitative PCR.

### Small interfering RNA transfection

Small interfering RNA (siRNA) targeting CXCL7 (Genomeditech, China), with the sequence provided in Supplementary Table 1, was used for transfection. U266 and RPMI8226 cells in the logarithmic growth phase were seeded into a six-well cell culture plate. The transfection complex was prepared by mixing 125 μL of RPMI 1640 medium (Gibco, USA), 3.75 μL of Lipo3000 reagent (Thermo Fisher Scientific, USA), and 10 μL of siRNA. After allowing the transfection complex to stand for 20 min, it was added to the six-well plate and gently mixed with the cell suspension. The plate was then incubated at the appropriate conditions for 24-48 h before changing to a fresh medium.

### Enzyme-linked immunosorbent assay (ELISA assay)

The concentration of human CXCL7 cytokine in the culture medium was measured using an ELISA kit (Abcam, England). All procedures were performed according to the manufacturer’s instructions.

### Quantitative RT-PCR analysis

Quantitative RT-PCR analyses were performed using the EZ Bioscience RNA Extraction Kit to extract RNA from control or treated cells, followed by cDNA synthesis with the Takara cDNA Synthesis Kit and real-time PCR using the Takara TB Green Premix Ex Taq (Tli RNaseH Plus) quantitative PCR kit. All primer sequences used in this study are shown in Supplementary Table 2.

### Western blot analysis

To lyse the cells, use 1X RIPA buffer containing Tris buffer (50 mM, pH 7.4), NaCl (150 mM), Triton X-100 (1%), PMSF (1 mM), SDS (0.1%), and protease inhibitor mixture from Beyotime Biotechnology. Cell lysates were mixed with Loading Buffer, separated by electrophoresis in a 10-15% SDS-PAGE gel, and transferred to a PVDF membrane from Millipore. Membranes were blocked with QuickBlock Blocking Buffer for Western Blot from Biotechnology. Membranes were washed three times with PBST and incubated with enzyme-linked secondary antibodies for 1 h at room temperature. Following PBST cleaning, luminescence was recorded with Lumin4000 from GE, USA, and ECL reagent from BioSharp. All antibodies used are listed in Supplementary Table 3.

### Subcutaneous Transplantation Tumor

CXCL7-OE and NC RPMI8226 cells were harvested during logarithmic growth, washed with PBS, and resuspended in the appropriate volume of PBS to achieve a density of f 5×10^6/100 μL. The cells were then subcutaneously injected into B-NDG mice within 2.5 h. Five randomly assigned 5-week-old female mice were divided into NC group and CXCL7-OE group. After alcohol disinfection, the mice were inoculated with 100 μL of cell suspension on the outside of the right upper limb and returned to their cages for further observation. The mice were monitored regularly for tumor growth, and tumor volumes were measured twice weekly using calipers. Mice were euthanized at the experimental endpoint, and tumors were excised for further analysis, including histopathological and molecular investigations.

### Xenograft tumor model in mice through intravenous injection

CXCL7-OE and NC RPMI8226 cells, harvested during logarithmic growth, were washed with PBS and resuspended in PBS at a concentration of 10^7 cells per 100 µL. The cell suspension was injected into the tail vein of 5-week-old female B-NDG mice within 2.5 h of preparation. Mice were randomly assigned into two groups: the NC group and the CXCL7-OE group, with five mice per group. Prior to injection, the tail veins were sterilized with cotton balls soaked in a 75% alcohol solution to promote vein dilation. A total volume of 100 µL of the cell suspension was injected in the direction of blood flow. Tumor growth and development were monitored regularly throughout the experiment. At the experimental endpoint, mice were euthanized, and tissues, including lungs, liver, and bone, were harvested for histological and molecular analyses.

### Immunohistochemistry (IHC) analysis

Mouse tumors were sampled and then embedded in paraffin and sectioned by a microtome. Immunohistochemistry was performed according to the steps provided by the manufacturer of the immunohistochemistry kit (Sevier, China), in which the primary antibodies used are also listed in Supplementary Table 3.

### Co-Immunoprecipitation (CO-IP)

To isolate the protein supernatant, cell lysates were subjected to centrifugation for 10–15 min. The resulting supernatant was incubated with the specific antibody overnight at 4 °C. Following this, the immune complexes were captured by magnetic beads through a 1-hour incubation. To eliminate nonspecific binding, the beads underwent three washes with IP buffer and one wash with distilled water. The immunoprecipitated proteins were then analyzed via WB.

### Co-culture of MM cells and mouse bone marrow-derived macrophages (BMDMs)

The co-culture system of MM cells and mouse BMDMs was established using a Transwell co-culture system. Logarithmically growing CXCL7-OE/NC RPMI8226 cells were seeded into the insert wells of the Transwell at a density of 1×10^5 cells per well, while BMDMs were plated in the lower chamber at a density of 5 × 10^5 cells per well. To extract BMDMs, 8-week-old C57BL/6 mice were euthanized by cervical dislocation following deep anesthesia. Both femoral bones were exposed and carefully cut open, and the bone marrow was collected using sterile scissors and forceps. The extracted bone marrow was placed into a dish containing sterile PBS, and the marrow was gently squeezed out using a syringe piston. The resulting cell suspension was filtered through a 70 μm cell sieve to remove debris and bone fragments. The filtered suspension was transferred into a culture flask with a complete medium, where it was allowed to settle for 4 h to remove non-adherent cells. The supernatant was then transferred into a new flask containing medium supplemented with M-CSF (2 ng/μL) and RANKL (20 ng/μL). After 6 days, osteoclast differentiation and morphology in the lower chamber were assessed using TRAP staining.

### Statistical analysis

Continuous variables were analyzed using Student’s t-test for normally distributed data or the Mann-Whitney U test for non-normally distributed data. PFS and OS probabilities were estimated using the Kaplan-Meier method, and differences in statistical significance were evaluated using the two-sided log-rank test. Categorical data were assessed using the Chi-square test. Statistical analyses were performed using R, version 4.3.3 (http://cran.r-project.org). A p-value less than 0.05 was considered statistically significant unless otherwise specified. All confidence intervals (CIs) were calculated at a 95% level. *P < 0.05, **P < 0.01 and ***P < 0.001 were noted.

## Reporting summary

Further information on research design is available in the Nature Research Reporting Summary linked to this article.

## Supplementary information

Supplementary Table 1

Supplementary Table 2

Supplementary Table 3

Supplementary Figure S1

Original Data File

Reporting Summary

## Data Availability

The single-cell RNA-seq processed gene expression data reported in this paper have been deposited into the CNGB Sequence Archive (CNSA) [31] of the China National GeneBank DataBase (CNGBdb) [32] with accession number CNP0005613(CSE0000445), and raw sequencing data have been deposited in the OMIX, China National Center for Bioinformation, Chinese Academy of Sciences (accession no. OMIX007530) [33]. To request access to raw sequencing data, please apply at the Human Genetic Resources Service System of the Ministry of Science and Technology (https://apply.hgrg.net/) in accordance with the Regulations on the Management of Human Genetic Resources of China. The multiple myeloma RNA-seq data GSE24080 were downloaded from the GEO database.
