# Supplementary material for: Myeloma cell-derived CXCL7 facilitates proliferation of tumor cells and occurrence of osteolytic lesions through JAK/STAT3 pathway
Source: Cell Death Dis. 2025 Feb 6;16(1):74. doi: 10.1038/s41419-025-07413-6 (PMC11802855; doi:10.1038/s41419-025-07413-6)
Supplement: Supplementary file 1 — Supplementary Table 1 [file 41419_2025_7413_MOESM1_ESM.docx]

**Supplementary Table 1. Sense primer sequences of CXCL7-siRNA.**

| siRNA | Sense primer （5’ →3’） |
| --- | --- |
| CXCL7(h)-siRNA-1 | GGAATTCATCCAAAAACA  UGUUUUUGGGAUGAAUUCC |
| CXCL7(h)-siRNA-2 | ATCCAAAGTTTGGAAGTGA  UCACUUCCAAACUUUGGAU |
