# Supplementary material for: Myeloma cell-derived CXCL7 facilitates proliferation of tumor cells and occurrence of osteolytic lesions through JAK/STAT3 pathway
Source: Cell Death Dis. 2025 Feb 6;16(1):74. doi: 10.1038/s41419-025-07413-6 (PMC11802855; doi:10.1038/s41419-025-07413-6)
Supplement: Supplementary file 2 — Supplementary Table 2 [file 41419_2025_7413_MOESM2_ESM.docx]

**Supplementary Table 2. Primer sequences used in the study.**

| Gene | Forward（5’→3’） | Reverse (5’→3’) |
| --- | --- | --- |
| GAPDH | GAAGGTGAAGGTCGGAGTC | GAAGATGGTGATGGGATTTC |
| CXCL7 | GTAACAGTGCGAGACCACTTC | CTTTGCCTTTCGCCAAGTTTC |
