# Supplementary material for: Myeloma cell-derived CXCL7 facilitates proliferation of tumor cells and occurrence of osteolytic lesions through JAK/STAT3 pathway
Source: Cell Death Dis. 2025 Feb 6;16(1):74. doi: 10.1038/s41419-025-07413-6 (PMC11802855; doi:10.1038/s41419-025-07413-6)
Supplement: Supplementary file 3 — Supplementary Table 3 [file 41419_2025_7413_MOESM3_ESM.docx]

**Supplementary Table 3. Antibodies used in Western blot and IHC.**

| Reagent | Purchasing Company | Catalog number |
| --- | --- | --- |
| Anti-CXCL7 Antibody | Abmart, China | PS08835 |
| Anti-JAK1 Antibody | Abcam, UK | ab133666 |
| Anti-p-JAK1 Antibody | CellSignalingTechnology, USA | 74129 |
| Anti-STAT3 Antibody | Abmart, China | T55292 |
| Anti-p-STAT3 Antibody | CellSignalingTechnology, USA | 9145 |
| Anti-CXCR2 Antibody | Abmart, China | TD7095 |
| Anti-MMP13 Antibody | Proteintech, China | 18165-1-AP |
| Anti-MMP2 Antibody | Proteintech, China | 10373-2-AP |
| Anti-MMP9 Antibody | Proteintech, China | 10375-2-AP |
| Anti-C-myc Antibody | Abmart, China | TA0358 |
| Anti-TRAP Antibody | Proteintech, China | 26846-1-AP |
| Anti-GAPDH Antibody | Proteintech, China | 60004-1-Ig |
| Anti-rabbit IgG，HRP-linked Antibody | Jackson, USA | AB_2338015 |
| Anti-mouse IgG, HRP-linked Antibody | Jackson, USA | AB_2338728 |
