# Supplementary figures and images for: Myeloma cell-derived CXCL7 facilitates proliferation of tumor cells and occurrence of osteolytic lesions through JAK/STAT3 pathway
Source: Cell Death Dis. 2025 Feb 6;16(1):74. doi: 10.1038/s41419-025-07413-6 (PMC11802855; doi:10.1038/s41419-025-07413-6)

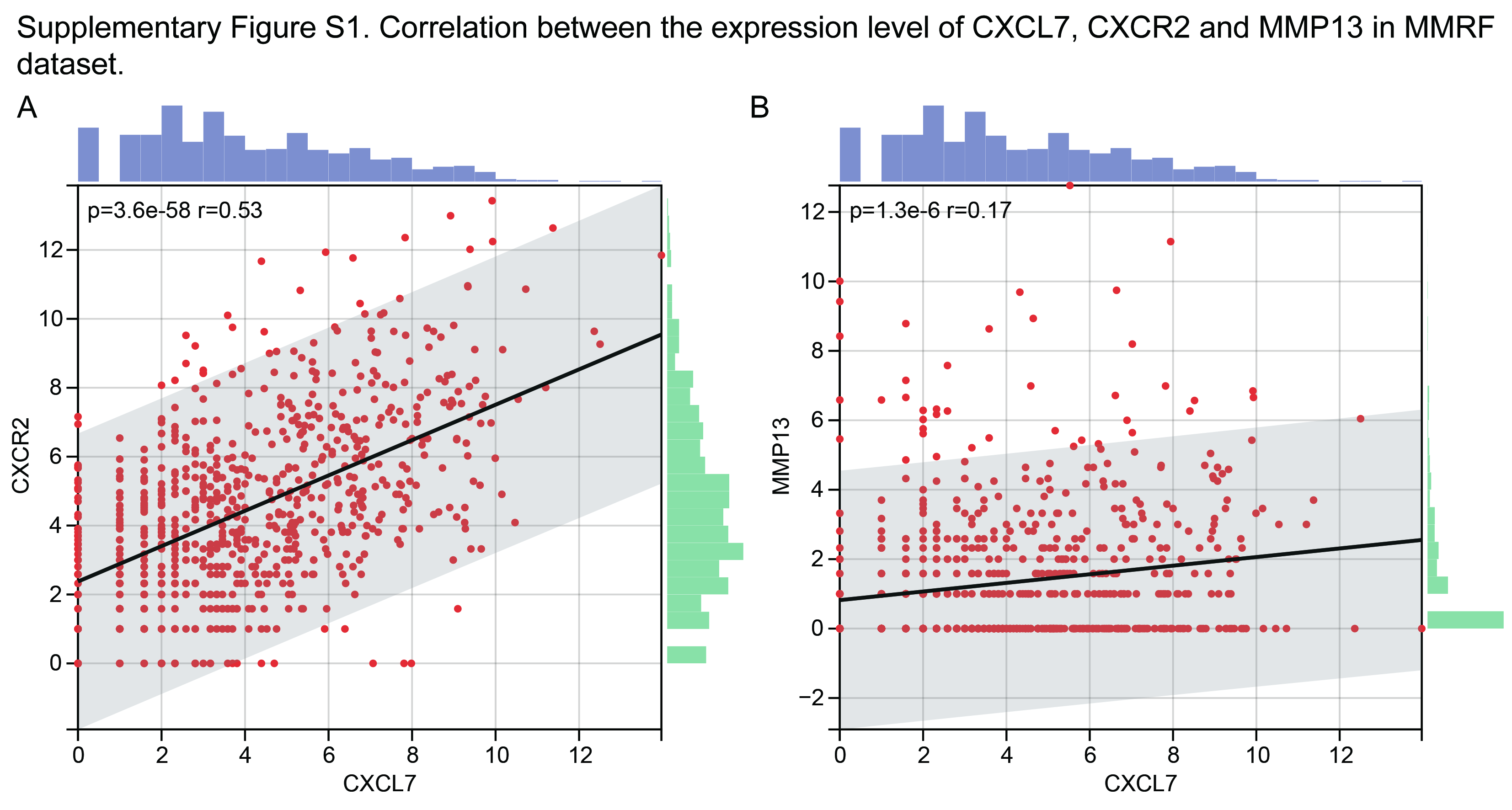

Supplement: Supplementary file 4 — Supplementary Figure S1 [file 41419_2025_7413_MOESM4_ESM.tif]
