# Supplementary material for: Myeloma cell-derived CXCL7 facilitates proliferation of tumor cells and occurrence of osteolytic lesions through JAK/STAT3 pathway
Source: Cell Death Dis. 2025 Feb 6;16(1):74. doi: 10.1038/s41419-025-07413-6 (PMC11802855; doi:10.1038/s41419-025-07413-6)

Figure 4f & 4g

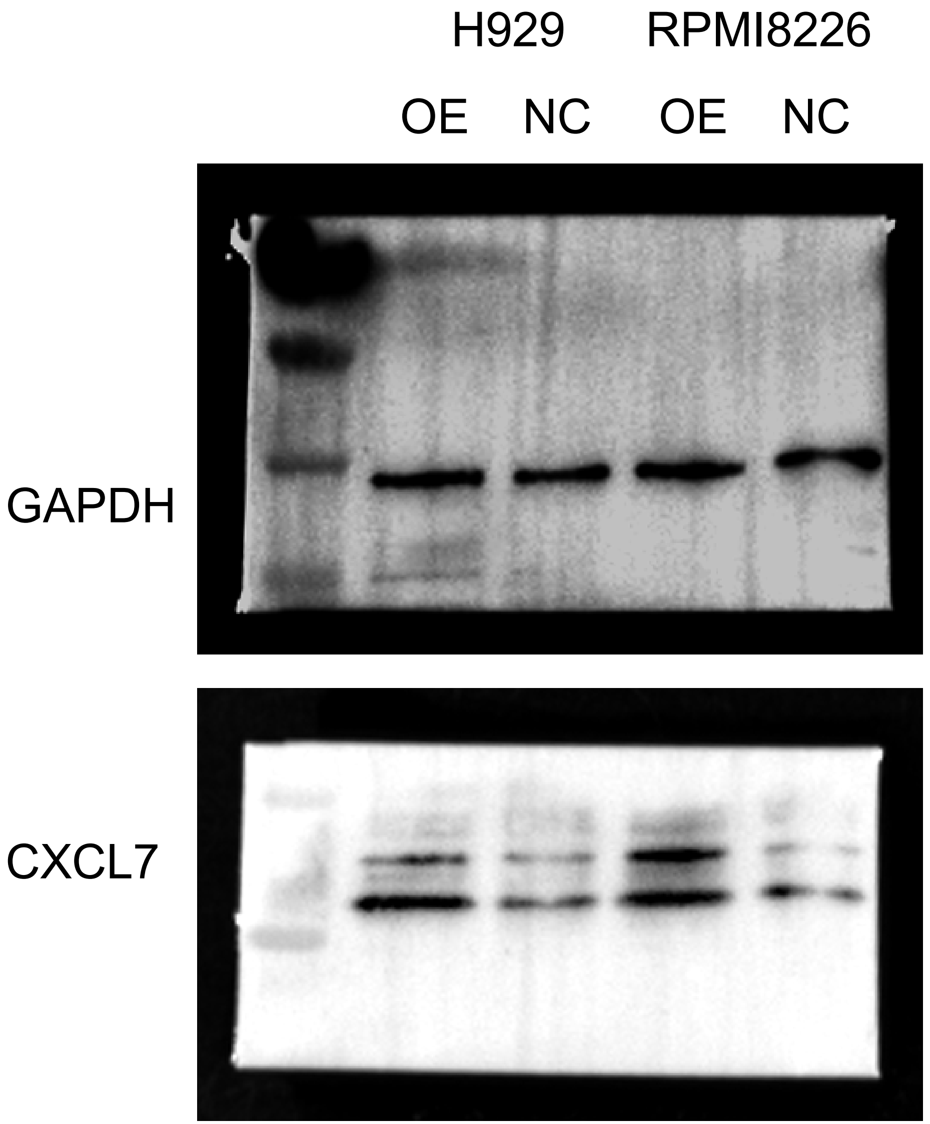

Figure 4h

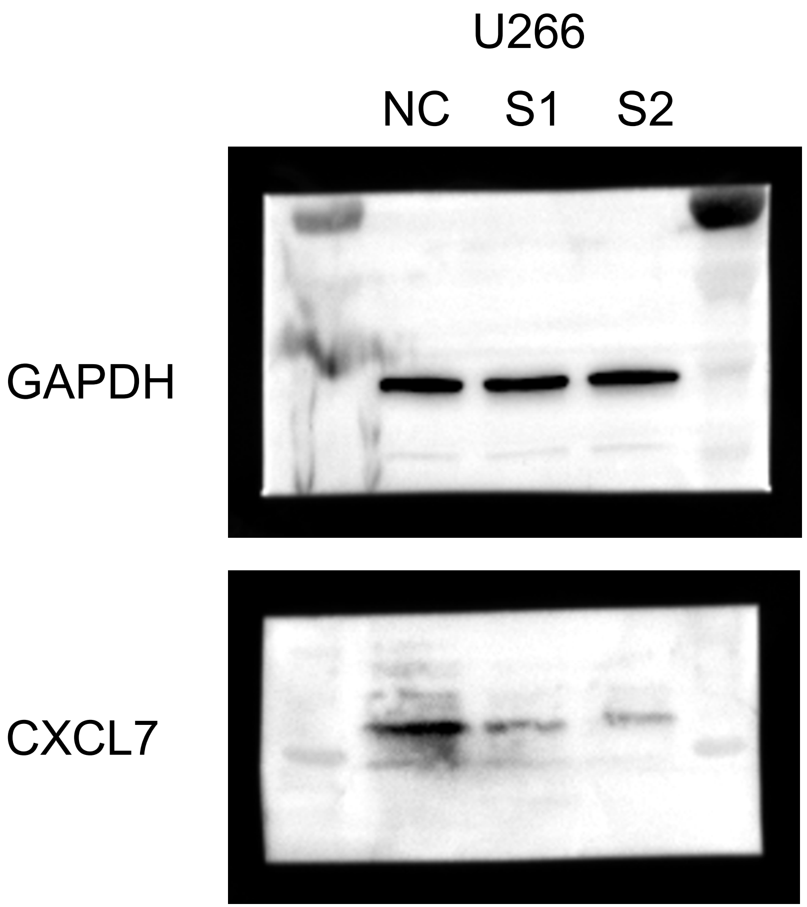

Figure 4i

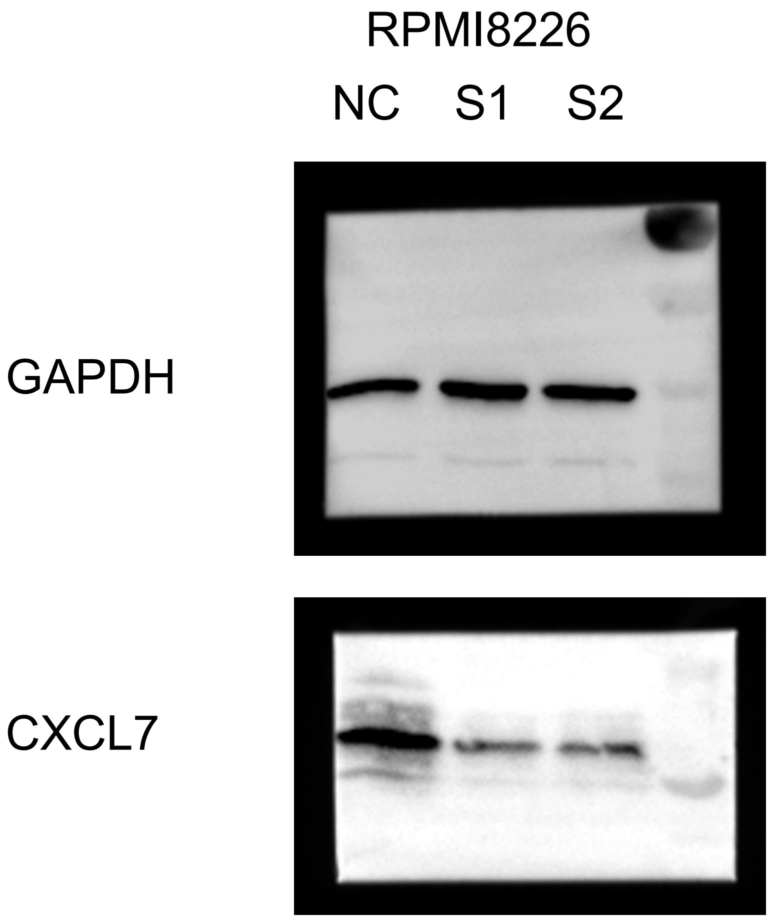

Figure 5e

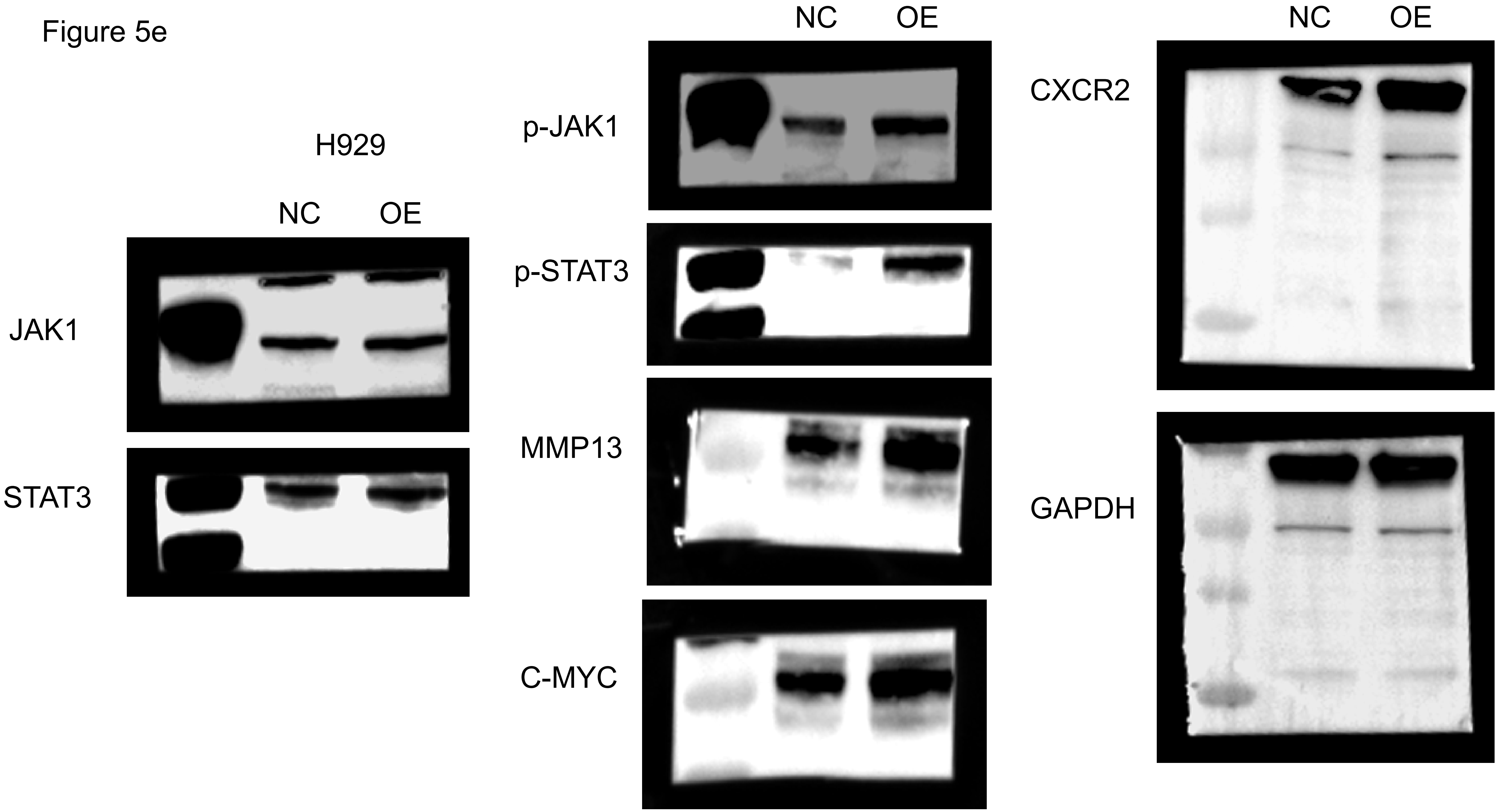
H929
NC
OE
JAK1
STAT3
NC
OE
p-JAK1
p-STAT3
MMP13
C-MYC
NC
OE
CXCR2
GAPDH

Figure 5e

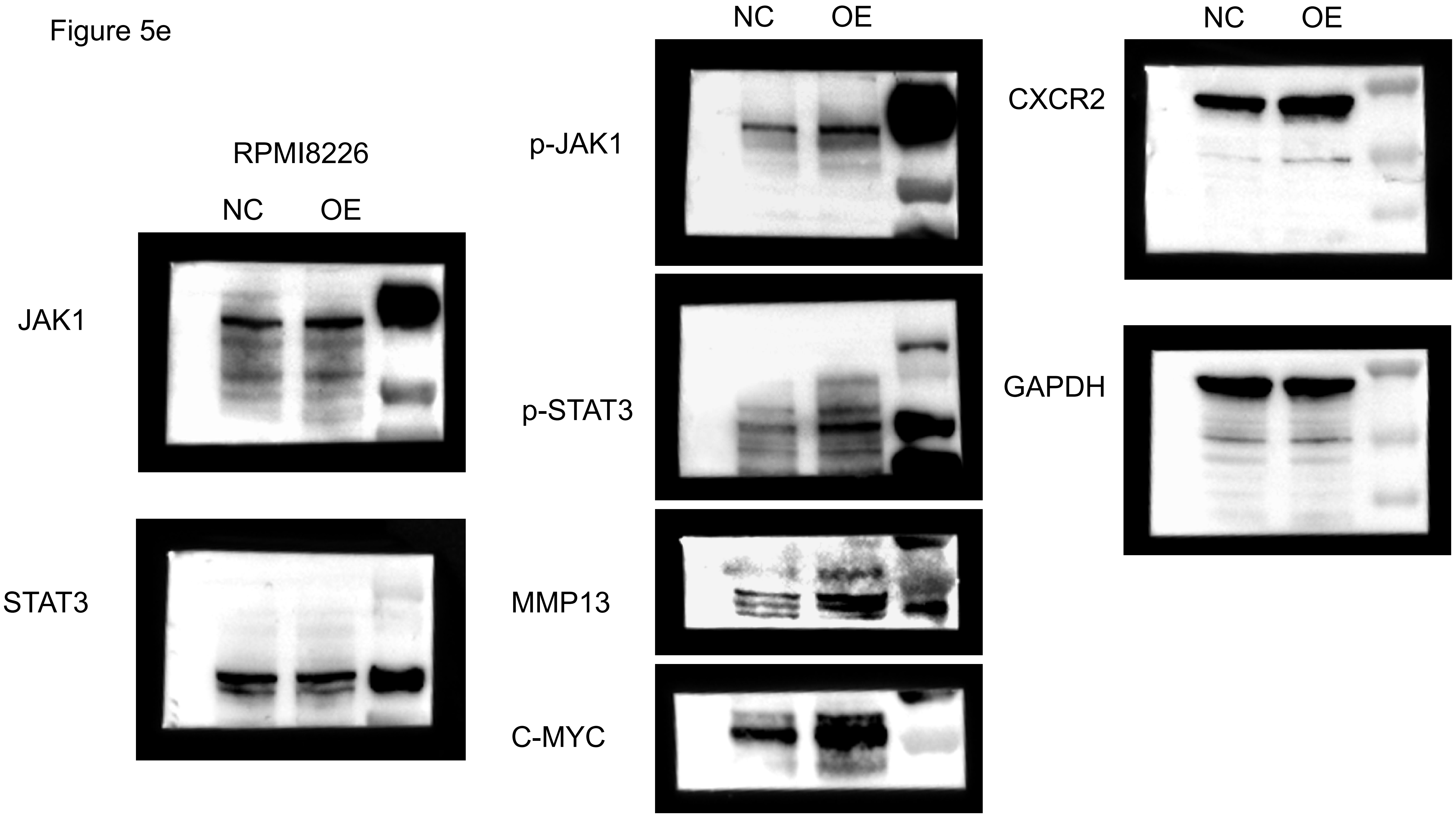
RPMI8226
NC
OE
JAK1
STAT3
NC
OE
p-JAK1
p-STAT3
MMP13
C-MYC
NC
OE
CXCR2
GAPDH

Figure 5f

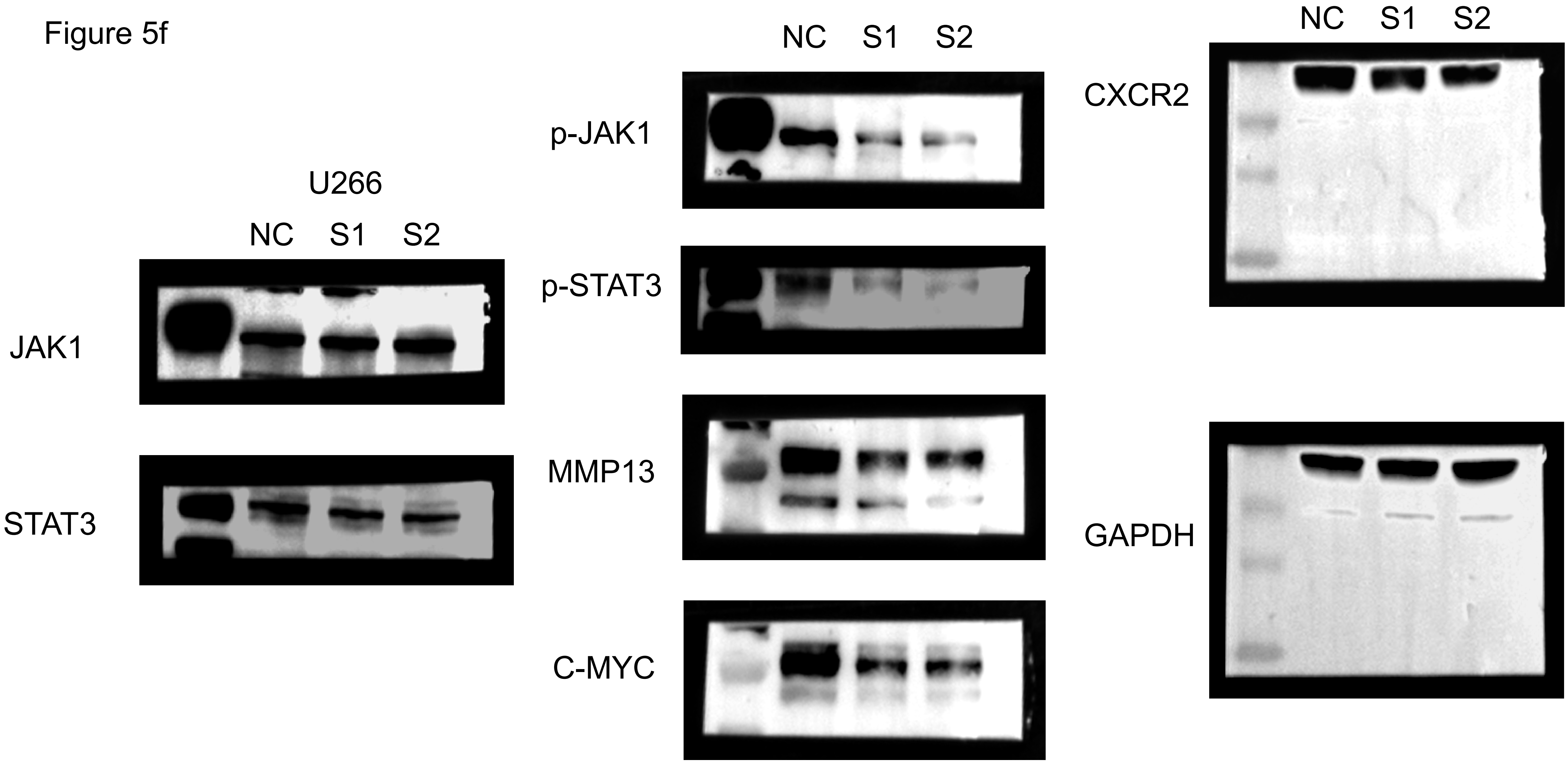
U266
NC S1 S2
JAK1
STAT3
NC S1 S2
p-JAK1
p-STAT3
MMP13
C-MYC
NC S1 S2
CXCR2
GAPDH

Figure 5f

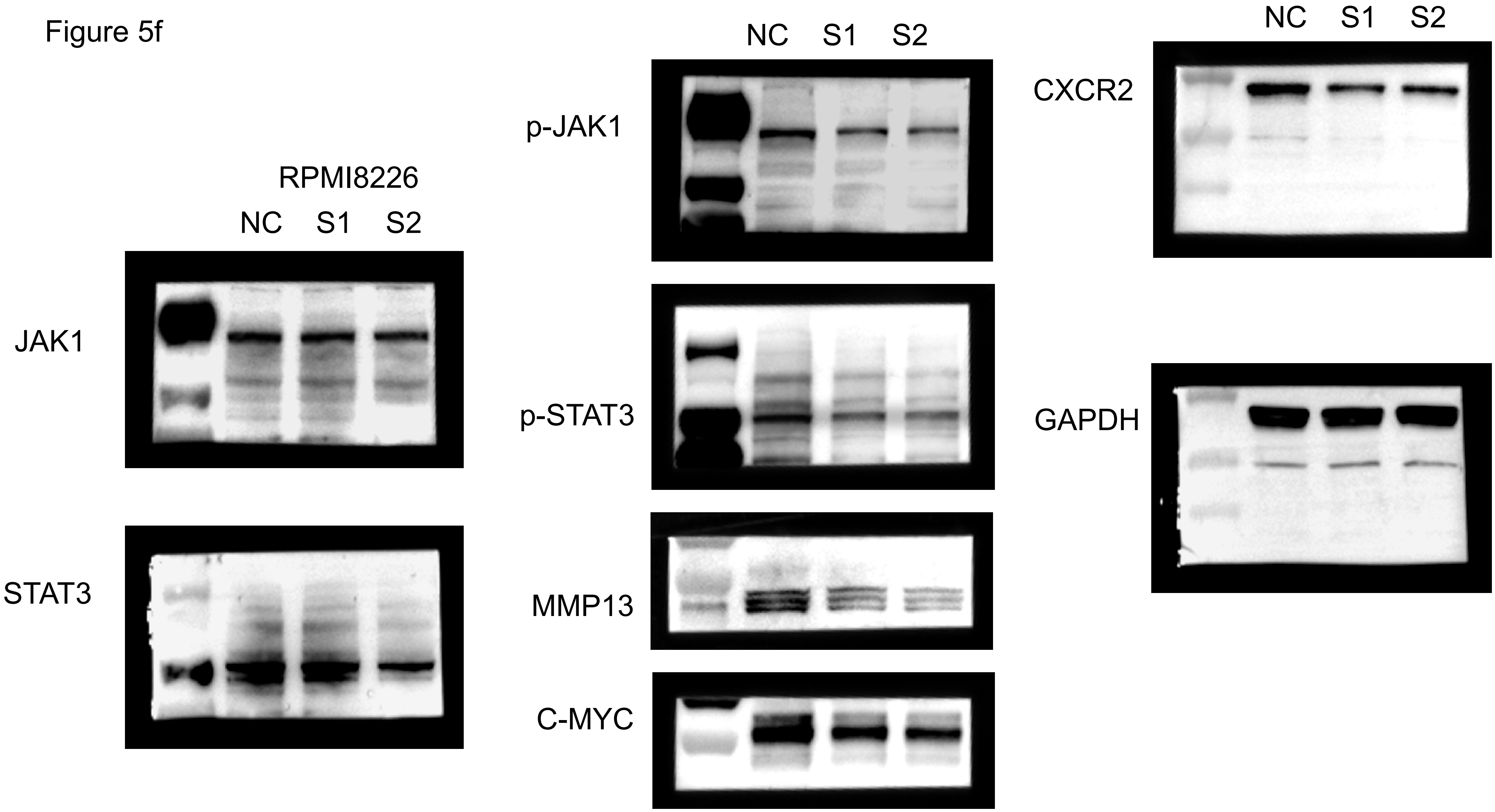
RPMI8226
NC
S1
S2
JAK1
STAT3
p-JAK1
p-STAT3
MMP13
C-MYC
CXCR2
GAPDH

Figure 5e

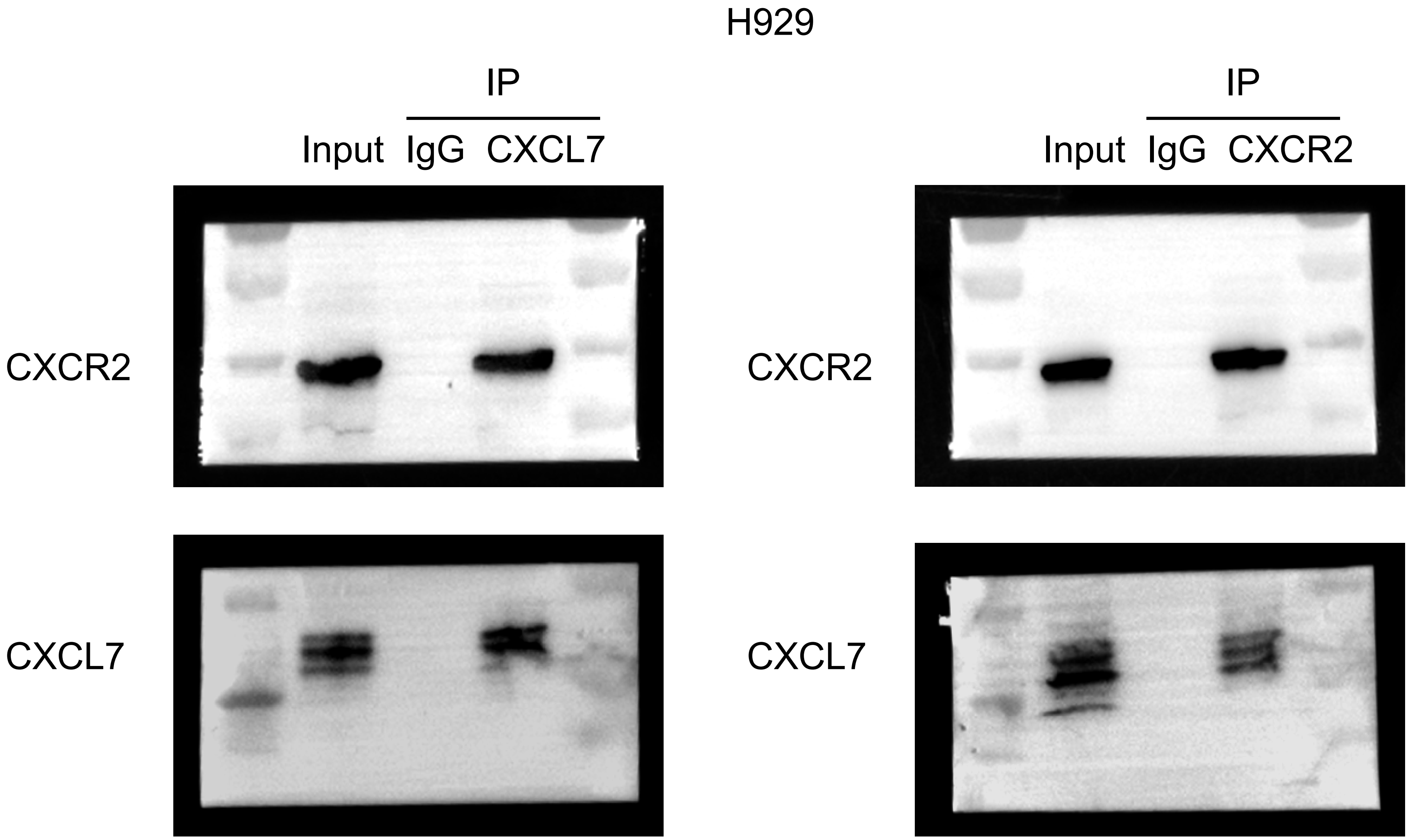
H929
IP
Input
IgG
CXCL7
CXCR2
CXCL7
IP
Input
IgG
CXCR2
CXCR2
CXCL7

Figure 5e

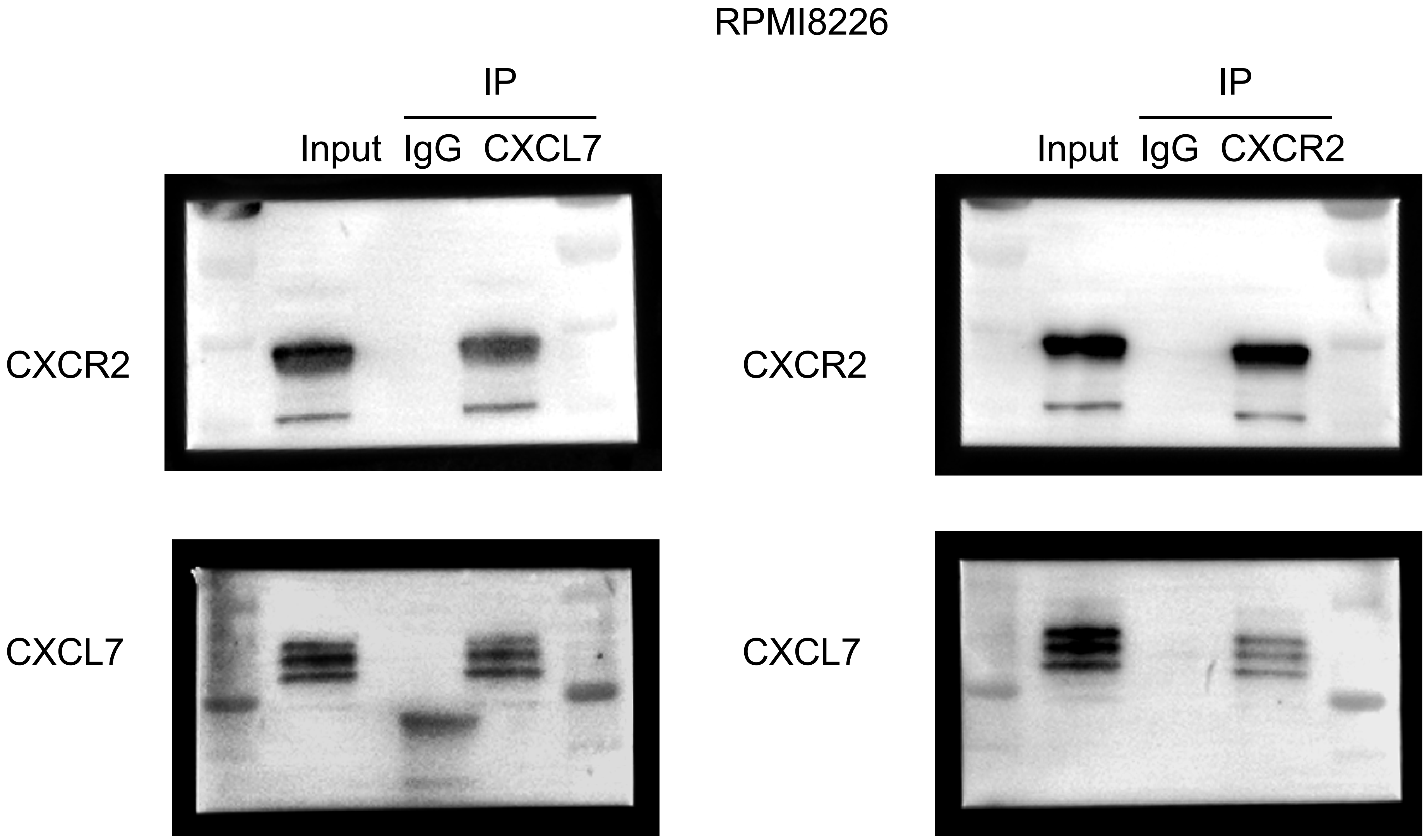
RPMI8226
IP
Input
IgG
CXCL7
IP
Input
IgG
CXCR2
CXCR2
CXCR2
CXCL7
CXCL7

Figure 7c

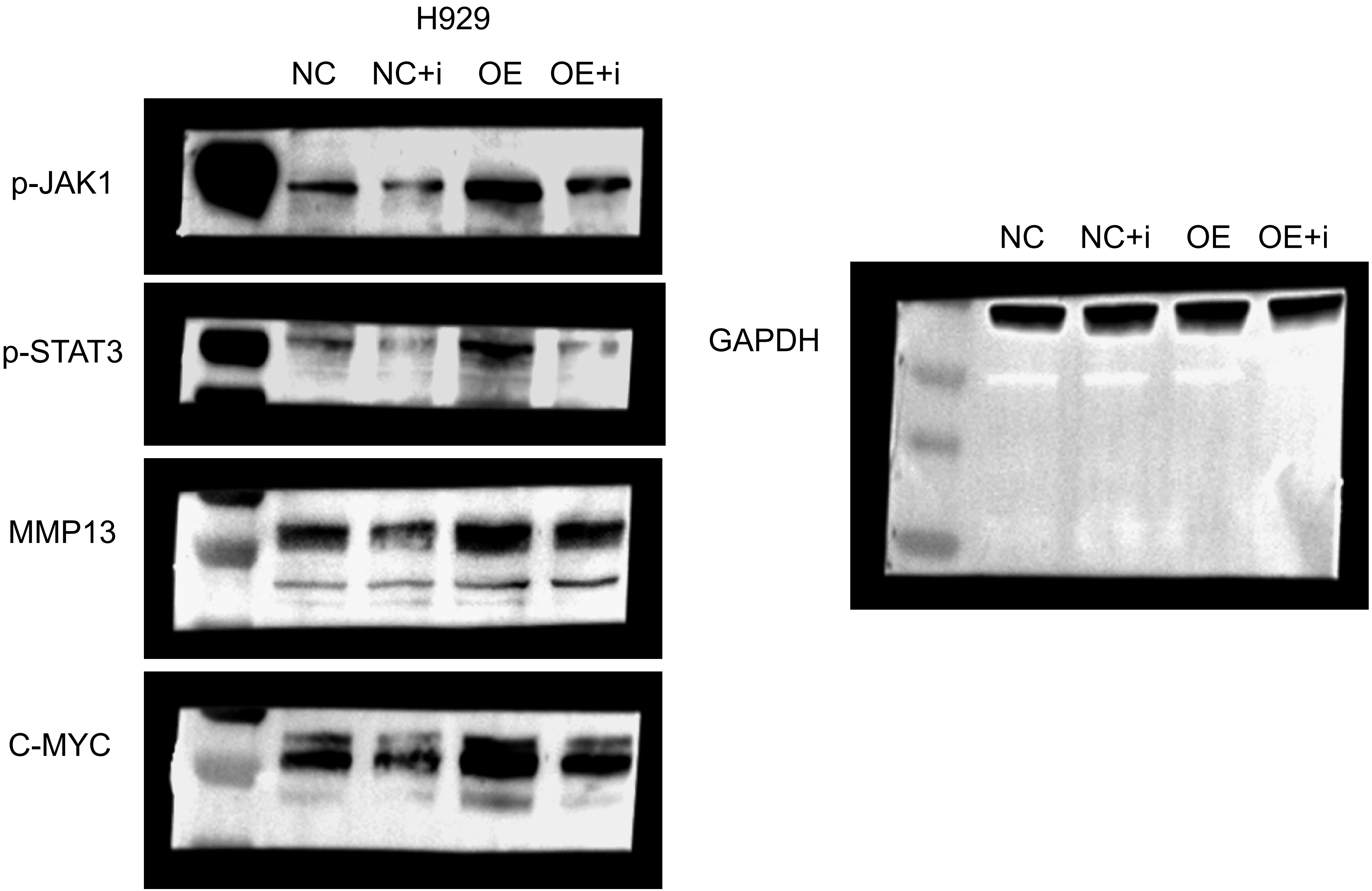

Figure 7d

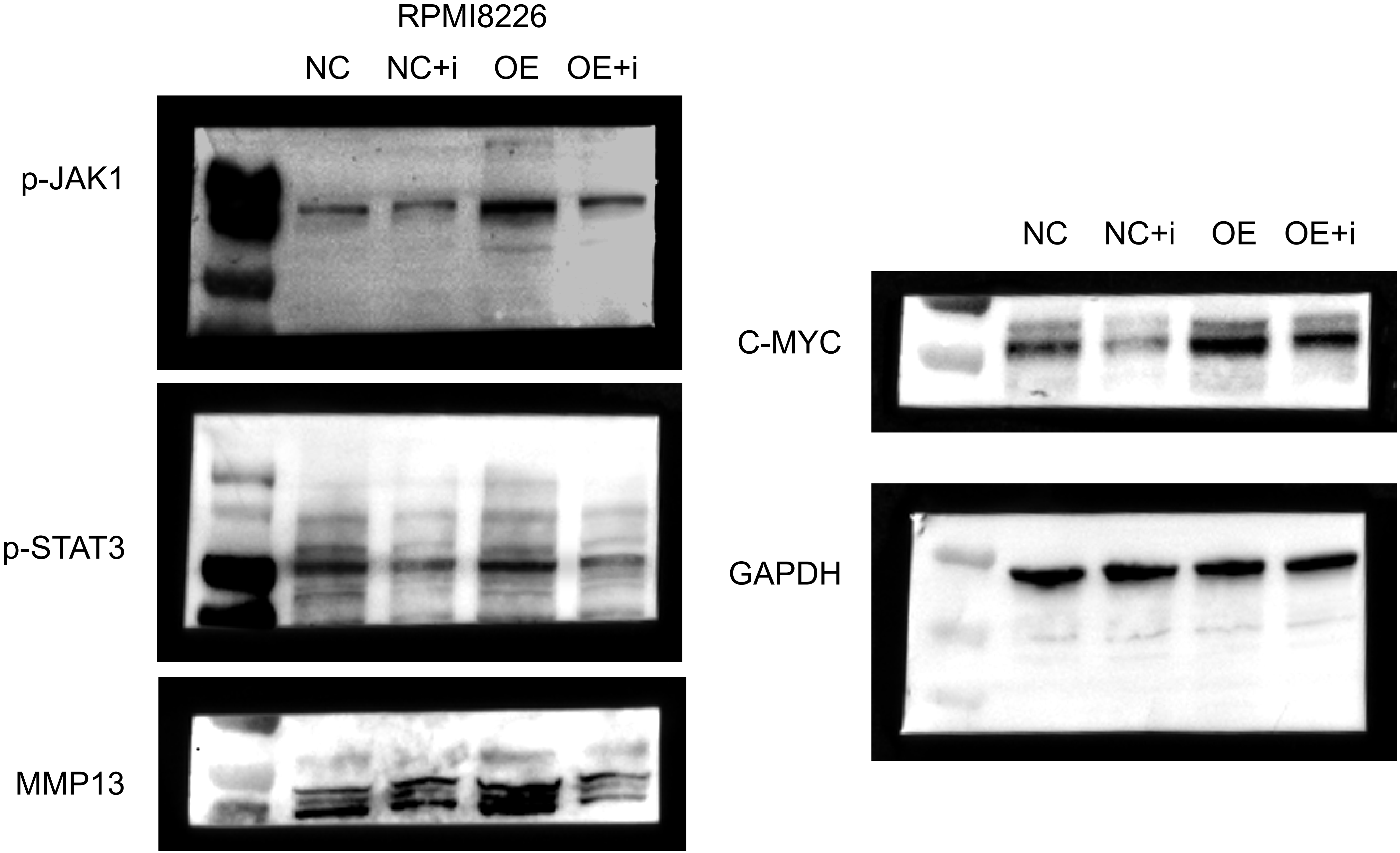
RPMI8226
NC
NC+i
OE
OE+i
p-JAK1
p-STAT3
MMP13
NC
NC+i
OE
OE+i
C-MYC
GAPDH

Figure 7e

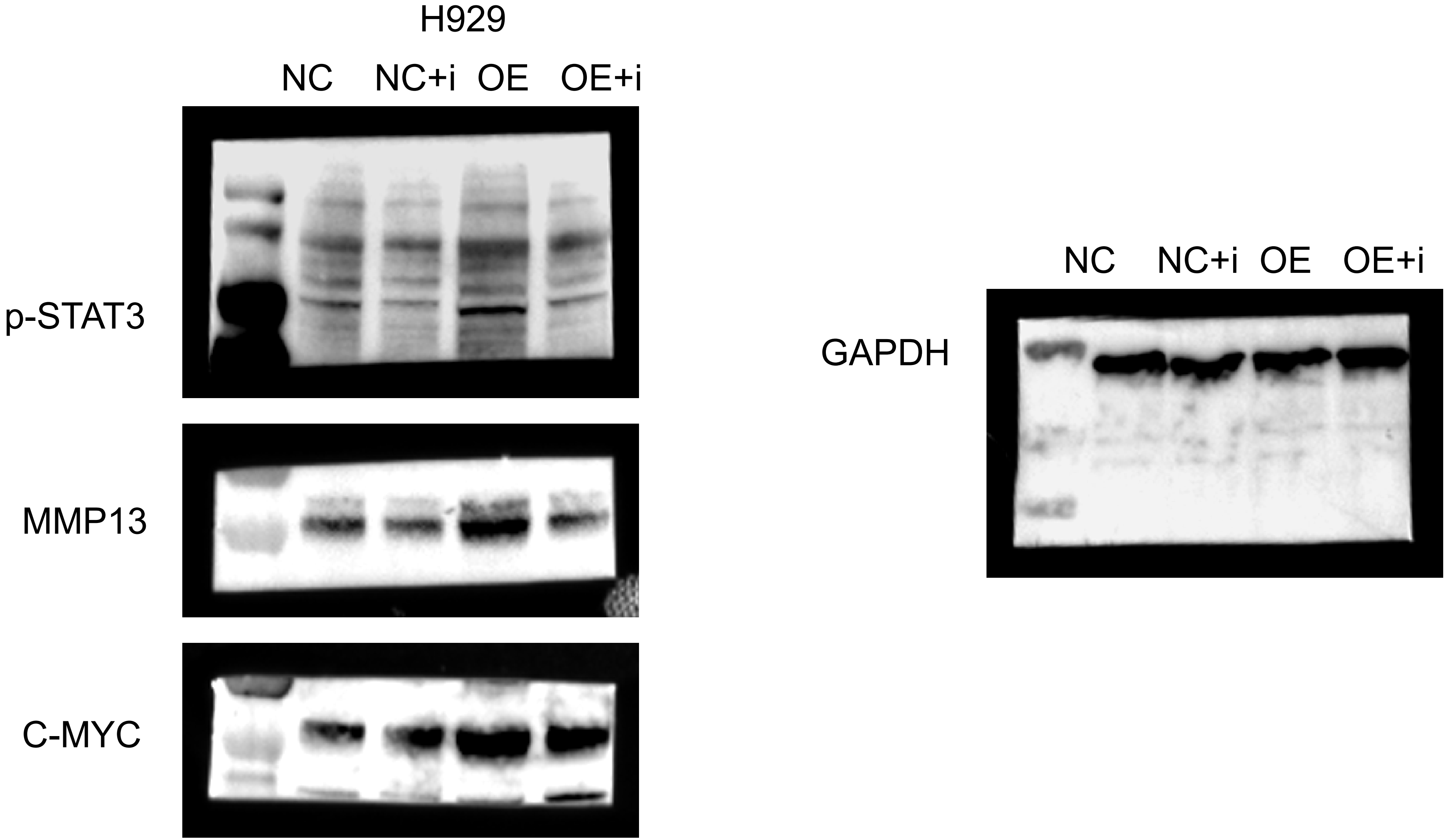

Figure 7f

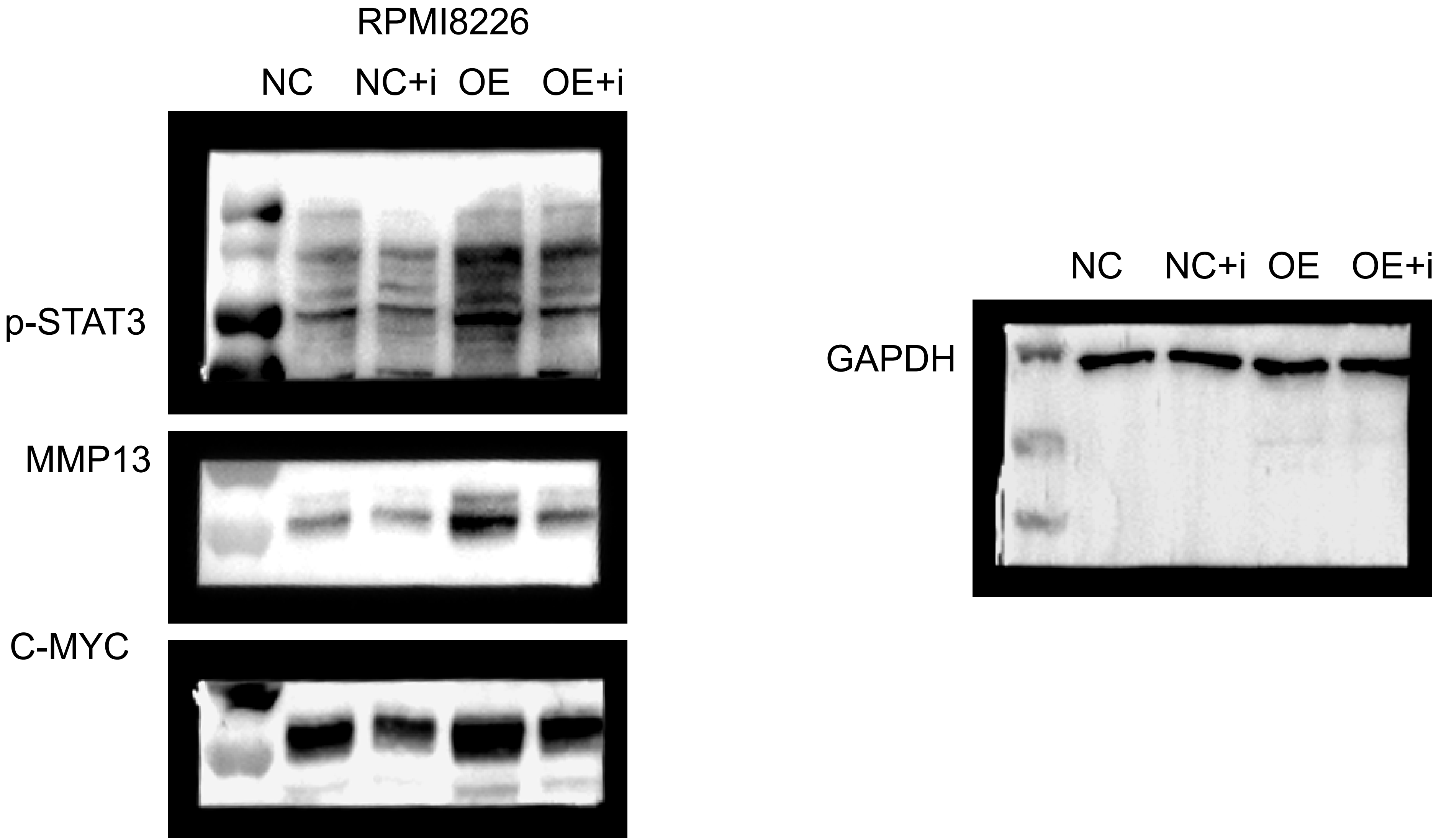

Supplement: Supplementary file 5 — Original Data File [file 41419_2025_7413_MOESM5_ESM.pdf]
